# Synthesizing the effects of mental simulation on behavior change: Systematic review and multilevel meta-analysis

**DOI:** 10.3758/s13423-021-01880-6

**Published:** 2021-05-04

**Authors:** Scott N. Cole, Debbie M. Smith, Kathryn Ragan, Robert Suurmond, Christopher J. Armitage

**Affiliations:** 1 Department of Psychology, York Saint John University, York, YO31 7EX UK; 2 Psychology, Leeds Trinity University, Leeds, LS18 5HD UK; 3 Psychology, Newcastile University, Newcastle upon Tyne, NE1 7RU UK; 4 School of Business and Economics, Maastricht University, 6229 GT Maastricht, Netherlands; 5 Manchester Centre for Health Psychology, University of Manchester, Manchester, M13 9PL UK; 6 Manchester Academic Health Science Centre, Manchester University NHS Foundation Trust, Manchester, M13 9PL UK; 7 NIHR Greater Manchester Patient Safety Translational Research Centre, Manchester, M13 9PL UK

**Keywords:** Mental simulation, Mental practice, Behavior change, Process simulations, Outcome simulations

## Abstract

Mental simulation of future scenarios is hypothesized to affect future behavior, but a large and inconsistent literature means it is unclear whether, and under what conditions, mental simulation can change people’s behavior. A meta-analysis was conducted to synthesize the effects of mental simulation on behavior and examine under what conditions mental simulation works best. An inclusive systematic database search identified 123 (*N* = 5,685) effect sizes comparing mental simulation to a control group. After applying a multilevel random effects model, a statistically-reliable positive effect of Hedges’ *g* = 0.49, 95% CI [0.37; 0.62] was found, which was significantly different than zero. Using a taxonomy to identify different subtypes of mental simulation (along two dimensions, class [process, performance, outcome] and purpose [whether an inferior, standard, superior version of that behavior is simulated]), it was found that superior simulations garnered more reliable beneficial effects than inferior simulations. These findings have implications for integrating theories of how mental simulations change behavior, how mental simulations are classified, and may help guide professionals seeking evidence-based and cost-effective methods of changing behavior.

**Supplementary Information:**

The online version contains supplementary material available at 10.3758/s13423-021-01880-6.

Imagining situations in one’s personal future is a common occurrence in humans (D’Argembeau et al., 2011), and recent experiments show that mental simulation can change a range of behaviors, including increasing fruit and vegetable consumption (Knäuper et al., 2011), improving accuracy on a pointing task (LaCourse et al., 2004), and increasing speed in a car-racing task (Callow et al., 2013, Experiment 1). Although there have been several attempts to review the literature on the effects of mental simulation on behavior change (e.g., Corbin, 1972; Driskell et al., 1994; Feltz & Landers, 1983; Richardson, 1967), these have typically been limited to unsystematic narrative reviews of the relatively narrow domains of “mental practice” or “mental rehearsal” that rely on prior experience of the target behavior, or have confounded mental simulation with other behavior change techniques. The aims of the present systematic review and meta-analysis are to address these limitations and discover (a) the unique effects of mental simulation and whether these effects are robust enough to hold across a range of domains and behavior types, and (b) under what circumstances mental simulation works best. In this way, this research harnesses the meta-analytic approach to examine the cross-disciplinary nature of a specific psychological phenomenon.

We believe there are four main reasons why this meta-analysis may have particular significance for behavior change research, and related fields (e.g., sports science, cognitive psychology) within the current literature.

First, within the behavior change literature, mental simulation has yet to be fully appreciated as a key behavior change technique in its own right, despite continued empirical research (e.g., Oh & Larose, 2015), and a recent systematic review and meta-analysis focusing on health interventions (see Conroy & Hagger, 2018). It may have received little attention because, despite some positive findings (e.g., Hagger et al., 2011), well-designed experiments in the field have resulted in nonsignificant findings (e.g., Conroy et al., 2015). This could lead to uncertainty among researchers as to the reliability and generality of the effects, perhaps due to the way in which mental simulation is typically conceptualized. For instance, in a taxonomy of 93 behavior change techniques developed by expert agreement, mental simulation was reduced to a subcomponent (15.2), labeled “mental performance of successful performance” (see Supplemental Materials of Michie et al., 2013). Although simulation of positive/optimal performance is an important aspect of mental simulation, the broader literature around mental simulation points to a plethora of subtypes that are used across diverse fields (i.e., inferior and superior; see Table 1). One aim of the current meta-analysis was to determine whether these subtypes substantially moderate the effects of mental simulation on behavior across varied domains—a question not addressed previously, and certainly not across a diverse range of studies.

**Table 1:** Possible subtypes of mental simulation

		Classes of mental simulation		
		*Process simulations*	*Performance simulations*	*Outcome simulations*
Purpose	*Inferior*	Simulating a plan that is ineffective	Simulating a performance that is below average	Simulating an undesired or feared outcome
	*Standard*	Simulating a standard/average plan	Simulating a standard/average performance	Simulating a standard/average outcome
	*Superior*	Simulating a plan that is effective	Simulating a performance that is above average	Simulating a desired or wished-for outcome

*Note.* Although there could plausibly be studies involving each subtype, some have yet to be studied. Nevertheless, here, we list some examples of the subtypes above from existing studies: superior process simulations (Pham & Taylor, 1999); inferior process simulations (no known example in existing literature); inferior performance simulations (Alden et al., 2001); standard performance simulations (Andre & Means, 1986); superior performance simulations **(**Callow et al., 2013); inferior outcome simulation (Marszał-Wiśniewska & Jarczewska-Gerc, 2016, Experiment 2); standard outcome simulation (no known example in existing literature); superior outcome simulation (Johannessen et al., 2012).

The second reason for conducting this systematic review and meta-analysis now is that several approaches and theories of episodic future thinking—the cognitive ability to mentally place oneself in a future context (see Atance & O’Neill, 2001)—have emerged in recent years. These theories draw upon findings from cognitive and neuroscientific research that are highly relevant to understanding the cognitive processes underlying behavior change resulting from manipulating mental simulation (see Baumeister et al., 2016; Schacter & Addis, 2007; Seligman et al., 2013). The current review aims to provide initial steps toward integrating more recent theoretical literature with meta-analyzed data, distinguishing fine-grained subclassifications of mental simulation (see below).

Third, the present meta-analysis improves on previous reviews by adjusting terminology to account for more recent literature adopting mental simulations. For example, when Driskell et al. (1994) published their meta-analysis, most mental simulation research emanated from the motor skill and sports domains. Driskell and colleagues’ definition of mental simulation was necessarily specific to these studies (restricted to “mental practice”/“mental rehearsal”), which often had highly scripted motor tasks (e.g., pointing tasks), physically practiced extensively before being mentally simulated. Recently, the term “mental simulation” has been used to simulate more varied behaviors relating to social behaviors (e.g., cooperation; Meleady et al., 2013) and education (e.g., studying; Pham & Taylor, 1999). Here, we adopted a broader conceptualization, defining mental simulation as mental representations of a behavior that can be hypothetical *or* familiar (practiced), and which can represent the future behavior in different ways, but in the context of the person not physically moving whilst simulating (Taylor et al., 1998). More specifically, we examined simulations of possible future scenarios (cf. “episodic future thinking”; Atance & O’Neill, 2001)—which could either be based directly upon a previous personal physical experience (i.e., familiar) or mentally simulated based on instructions from an experimenter, but with no direct experience of the scenario. This definition enables a meta-analysis such as this greater scope to capture the various ways researchers have studied mental simulation.

Finally, our meta-analysis utilized a sophisticated hierarchical or multilevel approach, which is useful in meta-analysis because, like with single studies (e.g., D’Argembeau et al., 2011), there may be multiple experimental and comparison groups. In prior studies, this dependency has gone untreated, simply assuming that all effect sizes were independent in these meta-analyses (Driskell et al., 1994). Alternatively, some have dealt with the increase in power when assuming independence by dividing the control group *N* by the number of conditions (Toli et al., 2016). However, this can result in a misleading increase in power (as univariate models count each study as independent). Thus, by including multilevel data, we model the dependency (Field & Gillett, 2010), ensuring our analyses are appropriate to the structure of the data.

## Types of mental simulation

In order to overcome limitations of previous mental simulation reviews, we explored the various forms of mental simulation in the literature. We summarize below the possible types and provide a breakdown of these along two dimensions (class and purpose; see Table 1). This is a conceptual structure aimed to help understand mental simulation and reduce the risk of confounding conceptually distinct forms of mental simulation.

To further distinguish mental simulations, it is known that they focus either on process, performance, or outcome. Process simulations involve imagining the procedural steps necessary to achieving one’s goal (e.g., “in order to run 5 miles every day, I will make sure I have the appropriate kit, book out time in my schedule, and jog to work in the mornings”; see Table 1 for a taxonomy of mental simulations). It is hypothesized that process simulations enable people to make concrete plans, developing a volitional action plan toward their goal (Taylor & Pham, 1996). Performance simulations are conducted when participants are asked to rehearse mentally or mentally practice a behavior after observing or performing that specific action (see Table 1). For example, Pascual-Leone et al. (1995) asked participants to repeatedly simulate a well-learnt five-finger piano sequence to improve precision and accuracy. Thus, performance simulations involve the participant mentally “running through” a specific task in chronological order, which is then tested behaviorally in a criterion task in identical way to the simulation. Outcome simulations involve envisioning a desired outcome (e.g., “I vividly imagine feeling relieved and satisfied having completed a marathon next year”), and are hypothesized to motivate people towards achieving their goal (Hagger et al., 2011; Pham & Taylor, 1999; Vasque & Buehler, 2007). Process, performance and outcome simulations can be carried out individually or in any combination and one of the aims of the present research is to tease apart in which combinations (if any) process, performance and outcome simulations differentially influence behavior. We shall call these different *classes* of mental simulation (see Table 1).

In addition, mental simulations differ on another dimension; how the achievement-level of the simulated behavior is framed—namely; (a) below-average (inferior) behavior, (b) average/unspecified behavior, or (c) above-average (superior) behavior. We called this the *purpose* of the mental simulation. The above-average *purpose* of outcome simulations (*superior outcomes*) can be manipulated through simulations of successfully completed behaviors, often associated with positive emotions and imbued with personal meaning (for similar concepts and interventions, see *approach goals*: Elliot & Harackiewicz, 1996; *possible selves*: Markus and Nurius, 1986, b; *fantasizing*: Oettingen, 2012). In contrast, *inferior outcome simulations* represent a poor outcome or complete failure to achieve one’s goal (for a similar concept, see *feared selves*: Markus and Nurius, 1986, b), but the latter may garner a motivational “incentive” as one may want to avoid this possible reality (see *avoidance goals*: Elliot & Harackiewicz, 1996). Each variant may trigger behavior change by highlighting discrepancies between current and possible future selves (Markus and Nurius, 1986, b). In our taxonomy, we define mental simulations based on what participants are explicitly instructed to imagine.

Additionally, studies considering the purpose of performance simulations have examined positive and negative simulations *of the criterion task itself*, with researchers expecting better behavioral outcomes following superior versus inferior performance simulations (Budney & Woolfolk, 1990; Woolfolk et al., 1985).

To date, process simulations have been studied in an undifferentiated way. That is, researchers define process simulations as functional or beneficial steps, which make goal attainment more likely (Pham & Taylor, 1999; Taylor & Pham, 1996). In Pham and Taylor’s (1999) words, “simulating the steps to reach a goal provides information about the actions needed to attain the goal” (p. 251). Although performance and outcome simulations can be positive or negative, few studies have examined steps that might be ineffective or counterproductive in goal behaviors. Nevertheless, we include superior, standard, and inferior process simulations in our classification, creating nine distinct potential subtypes of mental simulation (see Table 1). The aim in developing this taxonomy was to avoid redundancy and create orthogonal dimensions (i.e., class and purpose; see Hall et al., 1995, for a similar approach). In other words, it is reasoned here that any particular class of simulation (e.g., performance) can represent the future behavior in either a positive, neutral or negative way (see Table 1). Although this taxonomy is based on previous categorizations (e.g., positive versus negative outcomes; Marszał-Wiśniewska & Jarczewska-Gerc, 2016), these fine-grained distinctions are novel and have yet not been assessed as moderators in a meta-analysis. Further, we did not label inferior simulations as negative, and superior simulations as positive to avoid possible terminological confusion with affective simulations (see Gamble, Moreau, et al., 2019).

Previous reviews have tended to confound these different subtypes of mental simulation (e.g., Driskell et al., 1994; Feltz & Landers, 1983), thereby inappropriately aggregating various distinct categories into one abstract phenomenon. A recent meta-analysis has helped us to understand the effects of mental simulation in the domain of health interventions (and specifically, how intervention characteristics, such as follow-up simulations, moderate behavioral effects; Conroy & Hagger, 2018), but conceptually, simulations were only distinguished as process or outcome simulations (thus not addressing the effects of performance simulations). Finally, a meta-analysis on specificity of future thinking and depression, subdivided mental simulations based on their affective qualities (Gamble, Moreau, et al., 2019). We devised a taxonomy that was similar, yet distinctive, and that incorporated two dimensions (*class* and *purpose*; see Table 1), enabling us to incorporate subtypes from different fields and examining methodological characteristics involved in the effects of mental simulation.

## Methodological characteristics

### Measurement of task performance

Performance on any criterion task can be operationalized in a variety of ways, whether it is the speed with which a task is completed or how effective one is within a given time frame. More specifically, following simulation, a person may perform a task in a faster time, enact more behaviors over a set time, or hit a target with precision. It is plausible that the method of how one measures task performance itself may alter the relation between mental simulation and performance. Herein, these three ways of measurement are labeled *speed, frequency,* and *accuracy*, respectively. It is well known across psychology’s subdisciplines that physical practice enhances later performance—consequently, one will become *faster* on a specific task. One may predict similar facilitatory effects after mental practice. Mental simulation may increase the *frequency* with which a behavior is enacted by instantiating a “mental set”; one might physically enact a simulated behavior every time an appropriate opportunity arises, especially when the physical situation shares commonalities with the simulation (for a similar mechanism involving *implementation intentions*, see Gollwitzer, 1999). Epitomized by the phrase “practice makes perfect,” one may expect that simulation increases the ability to enact precise or accurate behaviors. Critically, although previous meta-analytic reviews (Corbin, 1972; Driskell et al., 1994; Feltz & Landers, 1983) examined type of task (cognitive vs. physical, reactive vs. self-paced), they did not go further in coding how behaviors are measured. Thus, our understanding of how mental simulation may differentially affect behavior as a function of outcome type is still weak. The current meta-analysis seeks to remedy this by examining its possible moderating effect(s).

### Dosage

One key moderator in medical trials is the frequency and duration of an intervention or its dosage (Higgins & Green, 2011). Establishing the minimal requirements for an effective mental simulation intervention is especially important here, where the cost-effectiveness of an intervention is often paramount (Hagger et al., 2011). Two points concerning dosage have emerged: Neither duration or frequency have a clear positive relation with effectiveness of mental simulation. First, long periods of mental simulation seem to reduce the benefits gained, despite an overall positive effect compared with controls (Driskell et al., 1994). The diminishing return of administering mental simulation over a long duration has been explained by a loss of interest (Driskell et al., 1994) and reactive inhibition (Corbin, 1972; see Feltz & Landers, 1983, for similar results and conclusions). Based on these findings, Driskell and colleagues recommended that mental simulation-based interventions be restricted to 20 minutes in duration. In a more recent meta-analysis, Conroy and Hagger (2018) found that, in health interventions, longer durations predicted more positive behavioral outcomes. Plausibly, the motor skill studies included by Driskell et al. (1994) contained studies with tens, hundreds, or thousands of simulation trials, leading to diminishing return effects. In contrast, health studies, the focus of Conroy and Hagger (2018), have far fewer simulation trials, on average (e.g., Hagger et al., 2011). Due to differences between domains sampled in previous meta-analyses, it was necessary to assess dosage across multiple domains here.

Concerning frequency, Feltz and Landers (1983) and Driskell et al. (1994) found no moderating effect of mental simulation upon the direction or size of the behavioral effect. However, Corbin (1972) theorized that there may be an “optimal” amount of mental simulation trials that lead to benefits on later behavior, after which, the beneficial effects decrease. This is reasoned to occur on the same grounds as *duration,* such as loss of interest in the simulation (after excessive numbers of simulation trials). However, theories of habit formation (for a review, see Carden & Wood, 2018) would suggest that repeated mental simulation might strengthen associations and lead to greater behavior change. We conducted comparable analyses of mental simulation duration and frequency in the present, broader meta-analysis. Such analyses will be necessary to infer general principles of mental simulation and provide guidance for future intervention design.

### Incentive

Payment, either by cash, course credit, or voucher is a common occurrence in psychological research, yet material reward is a behavior change technique in its own right (e.g., Michie et al., 2013) that influences performance on a variety of tasks/behaviors (e.g., Brase, 2009; Giuffrida and Torgerson, 1997). It would be valuable to see whether, for example, persistence with mental simulation tasks is influenced by the levels of material incentive offered for participation. This is important because payment to engage with mental simulation tasks would limit the widespread adoption of mental simulation in the wider world (e.g., in public health interventions).

### Delay

Delay (also termed retention interval or follow-up duration) is defined here as the time between the last mental simulation and the assessment of behavior. In their review, Driskell et al. (1994) examined 62 experiments and found that the mental simulation effect decreased linearly as the delay between the last mental simulation and outcome measure increased. Specifically, at 14 days, the mental simulation effect had reduced by half, and by 21 days it had fallen below the *d* < .10 typically denoting a small effect size (Cohen, 1988). Conroy and Hagger’s (2018) analysis of 24 control-simulation comparisons revealed no moderating effect of delay. It should be acknowledged that Driskell and colleagues did not include studies in domains such as health and social psychology, and Conroy and Hagger (2018) only assessed delay within health/social psychology, leaving a gap in meta-analytic data assessing across domains. We will attempt to reconcile these different findings to the broader grouping of mental simulation studies included here, while also including domain in the analysis (see below).

### Domain and behavioral task

One of the aims of the present review was to assess whether the mental simulation effect is transferable across, or is limited to, particular domains of study. Some authors of meta-analyses suggest that certain subtypes of mental simulation can enhance behavior (*motor planning*: Driskell et al., 1994; *health psychology*: Conroy & Hagger, 2018; *sports*: Feltz & Landers, 1983), whereas other studies in other domains have yet to be assessed within a meta-analysis (e.g., the effect of mental simulation within occupational or social domains). In the present review, we categorized studies in terms of their domain and examined whether each task had a largely cognitive, physical, or mixed (cognitive and physical) loading (i.e., Driskell et al., 1994; Feltz & Landers, 1983). Previous reviews indicated an advantage of mental simulation in more cognitive-based tasks as compared with physical-based tasks (see Driskell et al., 1994; Feltz & Landers, 1983).

### Experimental control and design

Finally, it is now recognized that it is difficult to obviate positively biasing those randomly assigned to experimental groups in psychological interventions because, in contrast to clinical trials involving medicinal interventions, participants are required to engage in psychological techniques (Boot et al., 2013). This issue may be prevalent in mental simulation research, as participants can reasonably “guess” they are in the intervention group. To reduce such effects, studies may include active control groups which involve a task, such as watching a video of an upcoming task. The present review will assess the extent of this issue by measuring studies with passive or active control groups. This will also address the issue of spontaneous engagement in mental simulation in control groups. Without active controls, or checks on mental activities of control participants, participants could be engaged in mental simulation, possibly to an equivalent degree to those assigned to intervention groups, thus reducing the potential benefit garnered by intentional mental simulation.

## Overall aims

Due to a confounded and fragmented literature, it was clear that a reappraisal of the mental simulation literature was warranted. This allowed a range of key moderators to be evaluated, such as outcome type, incentive, delay, domain, and task type (some of which have never been meta-analytically examined). Furthermore, as taxonomies are important in organizing knowledge, we developed a taxonomy of mental simulation subtypes. For the first time, this allows subtypes of mental simulation to be compared across studies in a new conceptual structure, which (1) better reflects the mental simulation interventions used, and (2) aids a clearer understanding of the possible mechanisms involved in the simulation process and its impact on behavior. It was envisioned that the present review could inform future research in applied (e.g., life coaching, health interventions) and scientific endeavors (e.g., understanding processes through which behaviors are modified): Less effective simulations can be deemphasized and optimal simulations more thoroughly investigated. This would have a two-pronged effect: Mental simulations could become regarded as a collection of behavior change techniques in applied psychology (Michie et al., 2013), and theoretical work into how and why mental simulation affects behavior would be energized (see Baumeister et al., 2016).

Based on previous work, we predicted that mental simulations (regardless of subtype) would exert larger behavioral effects where there is a longer intervention duration, participants were incentivized, and with shorter delay (Driskell et al., 1994; Feltz & Landers, 1983; Michie et al., 2013). We further explored whether subtype of mental simulation from our taxonomy would moderate effect sizes. Publication bias, and study quality (including manipulations checks) were also examined to assess any systematic biases in the existing peer-reviewed literature. The aims of the present systematic review and meta-analysis are, for the first time, to (a) synthesize the effects of mental simulation on behavior change across a range of domains, and (b) discover under what conditions mental simulation works best.

## Method

### Search strategy and process

In terms of selection, a systematic search of PubMed (including MEDLINE), PsychINFO, and Web of Science (including Arts & Humanities index; Science Citation Index Expanded; Social Science Citation Index) academic databases was conducted of all relevant English-language peer-reviewed articles published before May, 2020. Our definition of mental simulation incorporated its various forms, while maintaining a balance between sensitivity and specificity (see Cochrane Collaboration; Higgins & Green, 2011). The search terms consisted of descriptors of the intervention and outcome: (mental* AND rehears* OR simul* OR imagin* OR practi* OR prepar* OR possible sel* OR mental contrasting^1^) AND (behav* OR perform*) filtering to include peer-reviewed articles of human participants. To find randomized controlled trials, we included the search terms random* or experim*. In total, 4,161 articles were identified at the selection phase.

In the eligibility phase, abstracts were screened based on the following four criteria: Included experiments had to (1) have a between-group experimental design to which participants had been randomly allocated. Hence, only the most rigorous experiments (randomized controlled trials) were included, which provided a more stringent criteria than previous reviews (Conroy and Hagger, 2018; Feltz and Landers, 1983); (2) a control condition (passive or active) that only differed from the experimental condition through absence of mental simulation (e.g., mental simulation only versus passive control group; mental simulation plus feedback versus feedback only control); (3) an outcome measure that incorporated measurable behavior or performance (self-reported or objective); and (4) include a mental simulation intervention that was related to the main outcome measure. Studies were excluded from the review if they involved counterfactuals (past simulations) or memories, computer simulations, children (<18 yrs, because mental simulation ability changes through childhood; Skoura et al., 2009), older adults (>64 yrs, because age can affect mental simulation capacity; Zapparoli et al., 2013), or clinical groups (as the present review is concerned with “normal” cognitive-behavioral processes, and clinical disorders affect mental imagery (see Jeannerod, 1994, for a review). As we were interested in randomized controlled trials and because conference proceedings can introduce repeated data from various sources (and more dependency in the data), we decided to only include studies from peer-reviewed articles and test for publication bias thereafter. Specifying behavior as the main outcome measure as an inclusion criterion meant that experiments assessing outcomes only (e.g., body weight) were excluded unless they also measured the component behaviors (e.g., physical activity, avoiding snacks). In summary, our search strategy aimed to capture experimental studies from a variety of domains. In the abstract screening phase, the first author included or excluded experiments for the next stage using title and abstracts only. A subset of 10% of these were also categorized by the second author to check for reliability. This resulted in moderate to good agreement, as determined by Cohen’s kappa (85% agreement, Cohen’s kappa α = .58). All inconsistencies were resolved through discussion between the first and second author and then if a conflict remained, the final decision was made by a third coauthor.

**Fig. 1:**
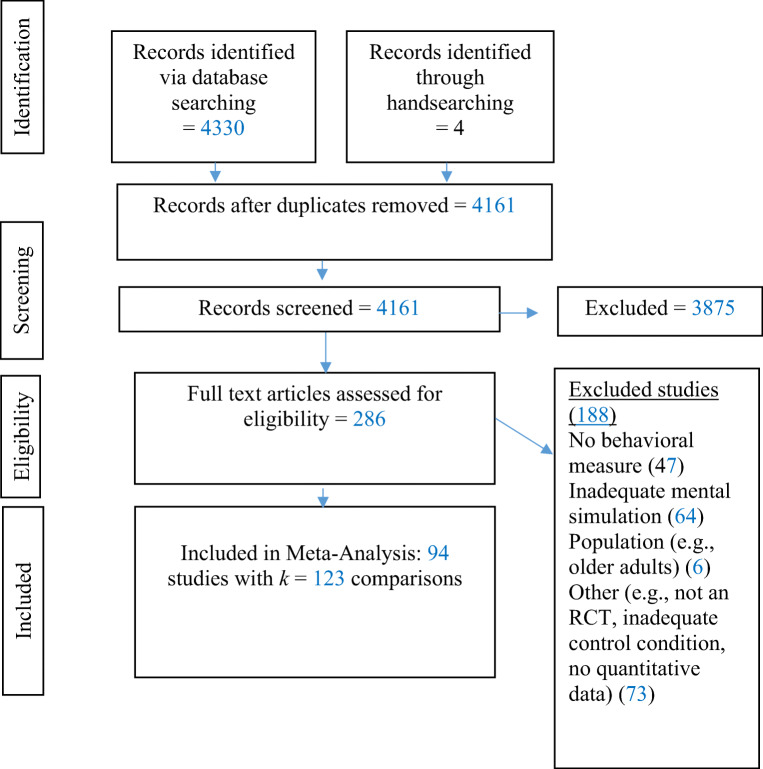
Flow diagram of systematic search results

**Fig. 2:**
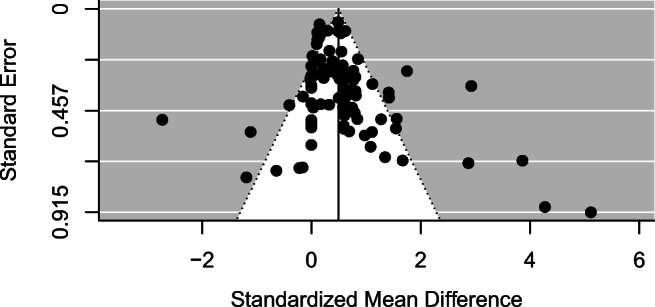
Main funnel plot for all included control-mental simulation comparisons (*k* = 123)

Thereafter, the full text of each experiment was examined by one coauthor to establish all relevant experiments that fit the review’s criteria (some included experiments which were derived from the same article, see Fig. 2). At this stage, the most frequent reason for exclusion was either the mental simulation manipulation not being tested uniquely (e.g., the mental simulation condition involved other additional components such as implementation intentions; e.g., Stadler et al., 2010) or an absence of a behavioral measure (e.g., body weight was measured; Lukaszewski & Jarczewska-Gerc, 2012, Experiments 5 & 6). Although fulfilling the criteria of this review, one experiment did not have sufficient quantitative data for inclusion in the meta-analysis (Bachman, 1990; an attempt was made to contact the author for further information). Finally, all coauthors agreed on the 94 studies (123 effect sizes) that were included in the following quantitative analyses, and the methods of this meta-analysis conformed to the PRISMA guidelines (see Supplementary Materials). We also conducted manual searches of the reference lists of all included articles retrieved from these searches to identify additional articles. This led to four additional articles being identified (a summary of search results is given in Fig. 1).

### Data extraction

In this phase, one coauthor (all trained on the coding extraction and mental simulation taxonomy) extracted data from included experiments included in the full text phase. To ensure high standards of data reporting and thoroughness, data extraction was based on guidelines and a proforma by the Cochrane Collaboration (see Higgins & Green, 2011), covering *N* size, sample characteristics, experimental conditions, outcome variables, quality (i.e., manipulation check of mental simulation and control), delivery method, and control condition. Some supplementary categories were added based on the moderator variables described above (e.g., incentive, delay between last mental simulation, behavior).

### Coding the subtype of mental simulation

As per the taxonomy specified in the Introduction, in which we identified nine distinct classes of mental simulation, varying along two dimensions; *class* (process/performance/outcome) and *purpose* (i.e., inferior/standard/superior), each experiment was categorized into one of these subtypes. The first author coded all studies, and a subset (at least 10%) were agreed with a second coder by consensus. There were no disagreements in the reviewed subset.

### Coding of the outcome variable

We also coded the outcome variable based on the assumption that mental simulation could affect behavior three main ways, *speed* (e.g., reaction time on a pointing task), *accuracy* (an all-or-nothing variable involving someone meeting a target or not; e.g., passing an exam), or *frequency* (the amount of times a behavior is recorded; e.g., amount of times a target is hit, amount of fruit consumed). For a full list of all included studies and their study characteristics, see Supplementary Materials. To ensure consistency across coauthors, a standardized form was used for all data extraction. Any disagreements between coauthors were resolved through discussion with the senior coauthor (C.A.) where necessary.

### Study and methodological characteristics

#### Coding of domains

We categorized the studies into the following domains: *health*, *social*, *occupational*, *sports*, *pain,* and *motor learning*. For the purposes of the present review, studies examining the mental simulation effect in *educational* contexts (e.g., Pham & Taylor, 1999) were classified as occupational due to the occupational requirement of students to learn.

#### Coding type of task

As in Driskell et al. (1994), we assessed the quality of the task employed in each study to assess whether mental simulation was more effective for tasks with either mainly cognitive or mainly physical attributes, or both. Cognitive tasks were defined as those that required mental operations, perceptual input, or decisions. Physical tasks were those that contained strength, endurance, or coordination. Mixed tasks were those that required both cognitive and physical components (definitions in line with Driskell et al., 1994).

#### Coding of dosage

Several metrics were used to determine dosage. As duration of mental simulation was recorded in minutes—studies in which the duration of mental simulation was estimated as “short,” but not further specified were assigned a value of 1 minute, except studies in which the duration of mental simulation was calculable. All mental contrasting interventions were given 5-minute duration by default (based on studies in which duration *was* stipulated; e.g., Marszał-Wiśniewska & Jarczewska-Gerc, 2016, Experiment 2), unless otherwise stated. Duration was coded into three categories: Short = 1 (1–5 mins), Medium = 2 (6–20 mins), Long = 3 (21+ mins). We also assessed the effect of frequency of mental simulation. Finally, we also multiplied length of (a single) mental simulation (e.g., 2 min) by the total frequency of mental simulations in the intervention (e.g., 5, thus total duration = 2 × 5 = 10).

### Methodological quality

First, we coded whether mental simulation was assessed in the control condition because without this measure, it is difficult to know whether, or the extent to which, participants spontaneously adopted mental simulation, minimizing the effect of any experimental manipulation (for example, after practicing a task, physically, one might spontaneously engage in mental simulation). Second, some studies ensured control participants were prevented from using mental strategies (for example, they were asked to carry out mental calculations or read a section of text). Thus, the subtype of control condition was also coded into two categories: either (a) a wait/passive control or (b) an active control that required considerable attention (e.g., verbal tasks, mental arithmetic, writing tasks). Third, studies were then coded for whether mental simulation was assessed in the experimental condition, to assess whether compliance was verifiable (e.g., reporting the contents of the simulation: Meslot et al., 2016; measuring the subjective vividness of the imagery: Kornspan et al., 2004).

Potential bias was assessed based on criteria from the Cochrane Collaboration (Higgins & Green, 2011) and Chambers (1990). Whether the methods incorporated (a) participant blinding, (b) experimenter blinding, (c) true randomization, (d) allocation concealment, and (e) outcome concealment was assessed as yes/no. In line with recommendations by Cochrane Collaboration (Higgins & Green, 2011), if no information was available on these aspects of bias, a separate code (“not described”’) was assigned. Hence, in this analysis, *k*s differed considerably depending on reporting and the codes assigned.

The first author coded all studies, and a subset (at least 10%) were agreed with a second coder by consensus. There were no disagreements in the reviewed subset.

### Meta-analytic strategy

Our meta-analysis required that we obtain a standardized mean difference (Hedges’s *g*) effect size (ES) for each control and mental simulation condition comparison. A positive ES indicated the degree of the mental simulation effect in favor of the experimental condition (a *g* of zero indicated no benefit, and a negative *g* represented a detrimental effect of mental simulation compared with a control group). As is typical in recent meta-analyses (e.g., Harkin et al., 2016), we corrected for small sample sizes by converting all effect sizes to Hedges’s *g* (Hedges & Olkin, 1985), which was used in all subsequent analyses.

The following descriptions apply to comparisons that had a continuous outcome variable. Where preintervention (baseline) and postintervention measures were reported, these were used to calculate pre-post change scores (by subtracting the pre from the post measure); otherwise posttest scores alone were used. To ensure conservative estimates, where multiple timepoints were recorded, only the farthest follow-up test was used to calculate the effect size.

Where means and standard deviation were not reported, but sample sizes were, we calculated Hedges’s *g* using the relevant *p* or *t* value. Where no comparison statistics were reported (such as an absence of means and standard deviations), study authors were contacted via email (*k* = 13, 11% of total studies), with three responding with data, four responding but with unavailable data, and six not responding. With no comparison data, the *p* value was assumed to be .05 when reported as “significantly different” and Hedges’s *g* = 0.00 when not (see Harkin et al., 2016, for a similar strategy). Where dependent variables were similar (e.g., binge and overall alcohol consumption; Hagger et al., 2012), an average was calculated. In addition, some studies measured qualitatively different outcome measures (e.g., speed and frequency), which may react differently to mental simulation. Where this was the case, up to three dependent variables (and effect sizes) were calculated per study to assess whether mental simulation is more effective for specific outcome measures.^2^ Where appropriate (e.g., duration not on target, for frequency of errors), dependent variables were recalculated (or reversed) so that *positive effect sizes always represented positive effects of mental simulation upon behavior versus a control condition* (e.g., duration on target, more efficient and effective behavior). Dichotomous data were converted into Hedges’s *g* by entering proportions of the control and treatment groups successful in the task using the online Practical Meta-Analysis Effect Size Calculator (https://campbellcollaboration.org/escalc/html/EffectSizeCalculator-Home.php [see “binary proportions”]) accompanying Lipsey and Wilson (2001). Heterogeneity of effect sizes was determined by the Q statistic and a significant heterogeneity indicates a need to uncover potential moderating effects.

We employed a multilevel meta-analysis to account for the dependency between effect sizes from one or more experiments from the same article (of the 94 studies included, 20, or 21%, contributed two or more effect sizes; all other studies contributed one effect size each). Using a univariate model would have forced us to drop observations and aggregate to one effect size per sample (or falsely treat each comparison as independent), whereas the multilevel approach allows us explicitly to incorporate this dependency into the model structure. In other words, multiple experiments are often embedded in a single study and therefore modeled as a multilevel structure using clustering through random coefficients. As appropriate in the social sciences due to the natural heterogeneity of findings, we apply a mixed-effects model that incorporates random coefficients (Overton, 1998; Viechtbauer, 2010). Random effects models are suited to meta-analyses that aim to generalize beyond the included studies in contrast to fixed effect models (Field & Gillett, 2010)—an aim held at project start. We also use inverse-variance weighting such that studies with more precision (usually those with larger samples) receive relatively more weight.

To explore the influence of moderators on the main relationship, we break the meta-analysis down into subgroups, including for different mental simulation subtypes and for other variables such as; outcome type, domain and methodological characteristics of the studies. This allows us to provide specific effect sizes for each of the subgroups and examine whether there are (significant) differences between these subgroups using a multilevel meta-regression approach. We use the metafor package for R to compute our models.

Although the protocol of this meta-analysis was not preregistered, the analysis script and data files are accessible via the open science framework (10.17605/OSF.IO/H2F7E) in line with the open science agenda (Munafò et al., 2017).

Publication bias was assessed by the statistical and visual inspection of a funnel plot (see Fig. 2). In this plot, the effect sizes (*x*-axis) from the studies are plotted against their standard errors (*y*-axis), and we find no evidence of asymmetry in this plot (asymmetry is assumed to indicate publication bias, where less precise studies—with smaller sample sizes and larger standard error—produce more positive effects). Additionally, we conducted an “Egger-style” analysis (Egger et al., 1997) by including the total sample size of the effects as a predictor in a meta-regression model. This approach allows us to maintain the integrity of the multilevel random effects model and still assess the influence of publication bias on our results directly. The results show that there is no relationship between the observed effects and the precision of the studies as estimated by the total sample size (*N* of experimental group and control group combined; β = −.001, *p* = .2284), and therefore there is no evidence that publication bias poses a major threat to the validity of our results.

Note that other, more traditional publication bias analyses are not applicable to the multilevel multivariate meta-regression model employed here. In particular, standard fail-safe numbers such as Rosenthal’s (1979) do not account for heterogeneity, and hence reflect a fixed-effect model that is deemed inadequate across most social sciences.

## Results

### Characteristics of included studies

The included studies yielded 123 independent effect sizes associated with mental simulation–control comparisons among 5,685 participants (control condition *n*s range 5 to 269; experimental condition *n*s range 5 to 181; see Supplementary Materials). The average age of participants was 24.97 (*SD* = 6.34), with the vast majority of studies using student samples (78%). The grand mean female:male participants ratio was 45:55 demonstrating a roughly even distribution across studies. Most behaviors were measured in terms of frequency of occurrence (*k* = 84); with the next common measuring speed (*k* = 35), then accuracy (*k* = 4).

### Overall effect of mental simulation on behavior

**Fig. 3:**
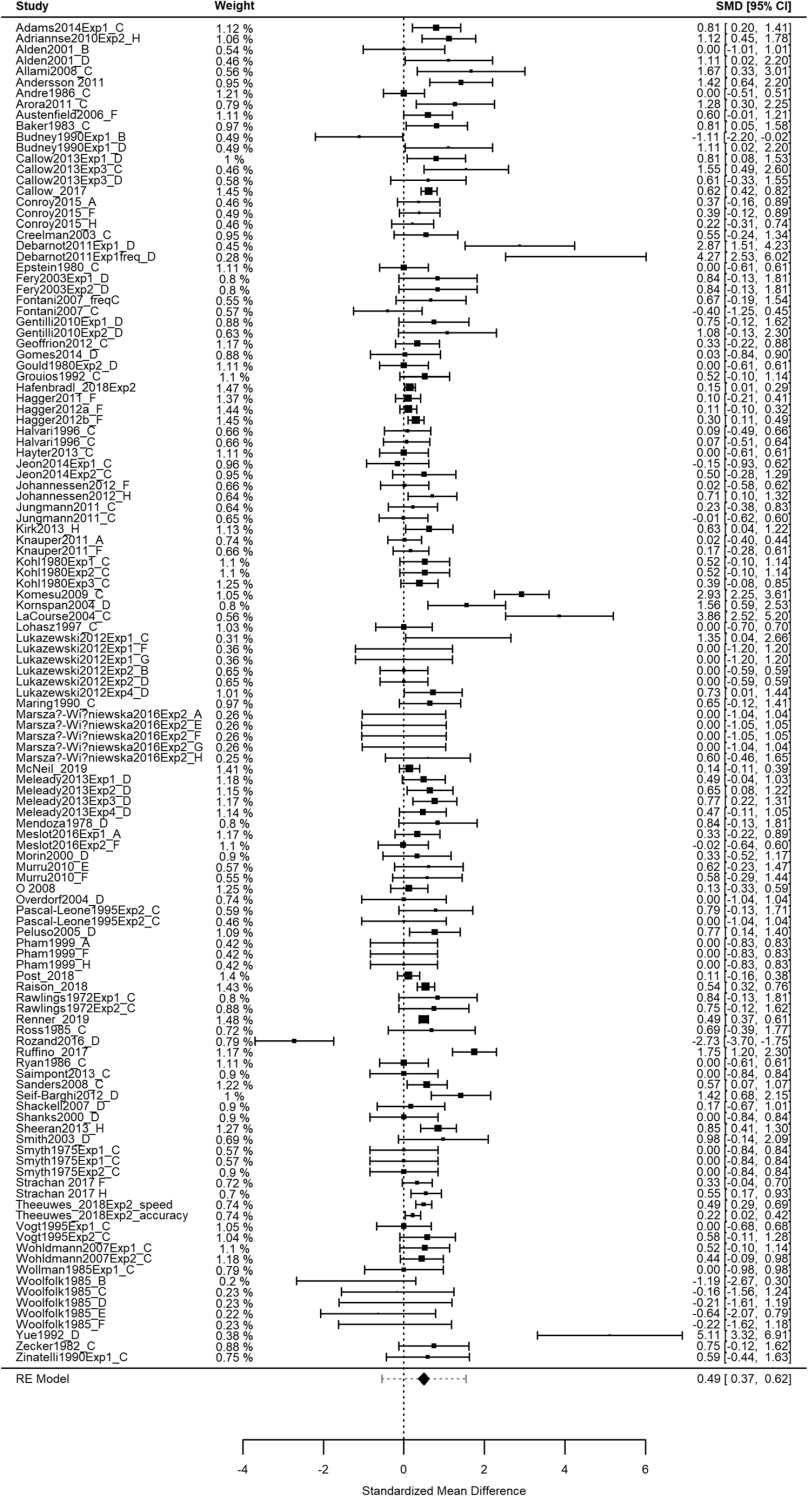
Forest Plot showing all 123 effect sizes included in the meta-analysis. Note for Figure 3: The vertical dotted line represents zero effect (mental simulation versus control). Each effect size (Hedges' g) is represented with a square, and all error bars represent 95% confidence intervals. The overall effect size presented as a diamond, with the width of the diamond referring to the 95% CI of that overall effect and the error bar to the 95% prediction interval. All means and confidence interval values are also presented on the right-hand side of each effect. Multiple types of mental simulation and dependent variables are shown independently, even if they are from within the same study. Weights are presented on the left-hand side of each effect, which reflect whether an effect is independent or is nested with other effects within the same experiment. Study authors and years are presented on the left-hand side and the type of mental simulation is represented by letters A-H (see Table 2 for descriptions of each simulation)

Here, and in all subsequent analyses, we applied a multilevel random effects meta-analysis in order to account for the dependency originated from several experiments conducted within one study (observable in Table 1). The weighted average effect of mental simulation upon subsequent behavior was Hedges’ *g* = 0.49 [0.37, 0.62]; *k* = 123 (94 studies), demonstrating an overall medium-sized positive effect of the intervention which was statistically significant, *z*(122) = 7.64, *p* < .0001. Overall, there was significant variability in effect sizes across the 123 comparisons (Q = 375.38, *p* < .001). Indeed, although the majority of studies found small-medium positive effects (as defined by Cohen, 1988), neutral (*g* = 0.00) and negative effects were also evident (e.g., *g* = −2.73; Rozand et al., 2016).^3^ A forest plot of all effect sizes (and the overall effect size) is represented in Fig. 3.

### Mental simulation subtypes

In coding the subtypes of mental simulation, some subtypes were more common than others. At least one study was found for each of the following subtypes: *process (standard)*, *performance (inferior standard, superior)* and *outcome (inferior, superior) simulations*. Additionally, it was found that some studies used composite subtypes, which were *inferior outcome plus process simulations* and *superior outcome plus process simulations*. No subtype overlapped two categories, and no studies were found in the literature utilizing *inferior process, superior process*, or *standard outcome simulations*. The full list of subtypes identified (with relevant illustrative examples) is presented in Table 2.

**Table 2:** Subtypes of mental simulation (and related examples) featured in interventions in the meta-analysis

MS class	Process	Performance			Outcome		Composites	
*Label*	Standard/unspecified (A)	Inferior/ below average (B)	Standard/unspecified (C)	Superior/above average (D)	Inferior (E)	Superior (F)	Inferior outcome + process (G)	Superior outcome + process (H)
*Number of effect sizes*	5	4	52	34	3	15	2	8
*Description*	Imagining the steps or plan involved in achieving a desired outcome (i.e., planning)	Imagining a performance that is below average	Mimic actual performance with no mention of effectiveness or efficiency of performance	Imagining a performance that is above average	Imagining an undesired or feared outcome	Imagining a desired or wished-for outcome	Simulation of an undesired outcome and simulation of ways to avoid this outcome	Simulation of a desired outcome and simulation of a barrier impeding the outcome
*Relevant behaviors within the context of the literature*	Imagining “subgoals” or “steps” toward a goal	Imagining “errors,” “being slow,” or “feeling uncomfortable”	Imagining same “responses,” “actions,” and “behaviors as actual performance	Imagining “perfect,” “fast” or “comfortable” performance	Imagining “receiving a low score” or “being unhealthy”	Imagining “breaking a habit,” or “getting a good grade”	Simulating a poor result on an attention task, and consideration of ways to avoid this outcome. (Lukaszewski & Jarczewska-Gerc, 2012, Experiment 1)	Mentally simulating a desired outcome with a potential barrier (Adriaanse et al., 2010).

We report the results for the weighted effect sizes of each mental simulation subtype separately in Table 3. When considering individual subtypes, it was found that the following mental simulations led to positive effects significantly different than controls: *Process*, *standard performance*, *superior performance*, *superior outcome* and *superior outcome combined with process simulations.* Mental simulations that included a negative purpose (*inferior performance, inferior outcome, inferior outcome combined with process simulations*) failed to demonstrate a reliable positive effect. While some subtypes appear to have different effects than others, an omnibus test of moderators shows that the effects are not statistically significantly different between subtypes of mental simulation, QM(*df* = 7) = 10.47, *p* = .1636. In other words, mental simulation appears to have a similarly large and positive effect on task performance across various subtypes. However, note that the number of effects included in several of these subgroups was very small (*k*), and reliable inferences cannot be drawn from those numbers.

**Table 3:** Effect size and heterogeneity as a function of mental simulation subtype

Moderator	*k (s)*	*g*	95% CI	QE	QM	*p*
Mental simulation subtype	123 (94)	–	–	342.25*	11.53	.12
Process (standard)	5 (5)	.17	[−.09, .43]	1.64		.20
Performance (inferior)	4 (4)	−.40	[−1.03, 0.22]	4.84		.20
Performance (standard)	52 (45)	.48	[.31, .65]	147.41*		<.0001
Performance (superior)	34 (33)	.67	[.34, 1.00]	161.94*		<.0001
Outcome (inferior)	3 (3)	.16	[−.51 .83]	2.41		.63
Outcome (superior)	15 (15)	.23	[.13, .34]	16.18*		<.0001
Combined (inferior outcome +process)	2 (2)	.00	[−.79, .79]	0.00		1.00
Combined (superior outcome + process)	8 (8)	.61	[.40, .81]	7.83		<.0001

*Note. k* = number of effect size estimates; *s* = number of studies; *g* = Hedges’s standardized mean difference; 95% CI refers to the lower and upper bounds of the 95% confidence interval around *d*; QE refers to the residual heterogeneity (Cochran’s Q), with * indicating that the probability of homogeneity of the data is less likely than *p* = .05; QM refers to the omnibus test of parameters for all moderators; *p* refers to the approximate *p* value of either the *z* value of the individual effect (*d*) or to the omnibus test of moderators (QM)

### Exploratory analysis of mental simulation purpose

As an exploratory analysis, we conducted an aggregated analysis of purpose (inferior, standard and superior), finding that superior and standard simulations were more effective than inferior simulations.

Some subtypes in the original analysis had only very few observations (inferior performance, *k* = 4; inferior outcome, *k* = 3; combined process and inferior outcome, *k* = 2). Here, we explain an exploratory analysis in which, due to lack of precision when examining the effect of mental simulation subtype, subtypes were aggregated into three instead of eight mental simulation subtypes. Mental simulation subtypes were collapsed into three main types: inferior, standard, and superior (referring to mental simulations with an inferior purpose such as imagining poor performance on a task; standard purpose, where the performance within the mental simulation was not specified; and superior purpose, in which participants are asked to imagine a good, optimal or best performance within their simulation).

Similar to the main analysis, a multilevel meta-regression using a random effects model was applied to investigate whether the effect of mental simulation on behavior is moderated by the “purpose” of the mental simulation itself (inferior, standard, superior).

The results suggest that the purpose of the simulation influences the effect is has on behavior. In particular, the meta-regression shows that the purpose aspect of the simulation explains part of the heterogeneity in effects (QM = 6.13, *p* = .047). The effect of inferior mental simulation is indiscriminate from zero (*g* = 0.01, 95% CI [−0.41, 0.44, *p* = .94). However, the effects of both standard and superior mental simulations are significantly larger (and positive): standard (β = 0.46, 95% CI [0.02, 0.91], *p* = .04) and superior (β = 0.53, 95% CI [0.11, 0.94], *p* = .014).

### Outcome measures: Speed, frequency, and accuracy

As outlined in the Method, we aimed to assess whether the type of outcome measure moderated the size of the effects of mental simulation on behavior. It was found that the type of outcome measurement did not significantly affect the mental simulation effect size. Regardless of whether the outcome measure was based on speed, frequency, or accuracy of behavior, a positive effect significantly above zero was found. Thus, mental simulation appears to have positive behavioral effects, regardless of the outcome type.

### Domain

The experiments were mostly derived from the motor skills domain (*k* = 49), and fewer were from health (*k* = 26), sports (*k* = 24), and occupational (*k* = 12) psychology. Far fewer studies investigated the effect of mental simulation on withstanding pain (*k* = 2) or social behaviors (*k* = 7). Domain was found not to be a significant moderator of the mental simulation effect in the omnibus test. Effects sizes significantly above zero were demonstrated in almost every domain except for pain, which did not significantly differ from zero (see Table 4). However, the lack of a statistical significance in this type of study should be taken with caution due to the limited amount of studies included here (*k* = 2), and the effect estimate is similarly positive.

**Table 4:** Moderator analyses

Moderator	*k (s)*	*g*	95% CI	QE	QM	*p*
Outcome	123 (94)	–	–	366.65*	.47	.7890
Speed	35 (31)	.373	[.137, .606]	118.77*	–	.0017
Frequency	84 (67)	.537	[.391, .683]	243.96*	–	<.0001
Accuracy	4 (4)	.329	[.030, .628]	3.93	–	.0310
Domain	123 (94)	–	–	374.00*	.31	.5774
Health	26 (16)	.371	[.241, .502]	41.67*	–	<.0001
Motor learning	52 (44)	.588	[.319, .856]	194.08*	–	<.0001
Occupational	12 (9)	.701	[.113, 1.289]	62.56*	–	.0195
Pain	2 (1)	.537	[−.551, 1.624]	2.15	–	.3334
Social	7 (7)	.434	[.197, .672]	10.36	–	.0003
Sports	24 (17)	.338	[.113, .562]	53.87*	–	.0032
Task	123 (94)	–	–	366.59*	1.04	.5934
Motor	52 (42)	.425	[.279, .571]	108.25*	–	<.0001
Cognitive	20 (16)	.313	[.161, .465]	21.99	–	<.0001
Mixed	51 (45)	.569	[.320, .819]	236.35*	–	<.0001
Duration (coded)	115 (88)	–	–	362.84*	0.05	.8204
Short	88 (71)	.480	[.354, .607]	252.03*	–	<.0001
Medium	16 (11)	.794	[.049, 1.539]	105.20*	–	.0368
Long	11 (6)	.481	[.315, .647]	5.25	–	<.0001
Frequency	123 (94)	–	–	374.75*	1.17	.2800
Total Time (Duration × Frequency)	123 (94)			374.93*	1.01	.3147
Delay	112 (87)	–	–	308.37*	0.08	.7679
Incentive	49 (33)	–	–	63.23	2.48	.1151
with incentive	41 (30)	.368	[.273, .464]	62.13*	–	<.0001
no incentive	8 (3)	.061	[−.276, .397]	1.10	–	.7247
Post-only or pre–post change scores	122 (93)	–	–	372.35*	0.34	.5582
Change scores	43 (30)	.455	[.163, .747]	173.34*	–	.0023
Post-only scores	79 (64)	.499	[.368, .630]	199.01*	–	<.0001
Participant blinded	29 (26)	–	–	130.18*	0.53	.4673
Blinded	26 (23)	.599	[.331, .868]	124.93*	–	<0.0001
Nonblinded	3 (3)	.833	[.317, 1.350]	5.25	–	.0016
Experimenter Blinded	23 (19)	–	–	111.71*	0.72	.3948
Blinded	14 (10)	.382	[.118, .647]	36.54*	–	.0047
Nonblinded	9 (9)	.718	[.125, 1.312]	75.18*	–	.0177
Randomization	41 (31)	–	–	175.32*	3.05	.0807
Randomized	23 (16)	.612	[.276, .948]	128.01*	–	.0004
Nonrandomized	18 (15)	.176	[−.171, .523]	47.30*		.3214
Allocation Concealment	17 (13)	–	–	93.64*	0.06	.8044
With alloc concealment	13 (9)	.727	[.124, 1.329]	84.58*	–	.0181
No alloc concealment	4 (4)	.571	[−.059, 1.200]	9.05*	–	.0755
Mental imagery in control	123 (94)	–	–	366.28*	3.63	.1626
With imagery check	20 (16)	.311	[.141, .481]	30.12	–	.0003
Without imagery check	98 (77)	.545	[.392, .697]	334.76*	–	<.0001
Mental imagery in experimental	123 (94)	–	–	374.06*	0.68	.4107
With imagery check	70 (51)	.457	[.262, .651]	259.24*	–	<.0001
Without imagery check	53 (43)	.482	[.351, .613]	114.82*		<.0001

*Note.* alloc = allocation; *k* = number of effect size estimates; *s* = number of studies; *g* = Hedges’s standardized mean difference; 95% CI refers to the lower and upper bounds of the 95% confidence interval around *d*; QE refers to the residual heterogeneity (Cochran’s Q) with * indicating that the probability of homogeneity of the data is less likely than *p* = .05; QM refers to the omnibus test of parameters for all moderators; *p* refers to the approximate *p* value of either *the z* value of the individual effect (*d*) or to the omnibus test of moderators (QM)

### Type of task

Experiments were divided into those that contained mainly cognitive or physical attributes, or a combination of both, to assess the type of the task that participants engaged with. However, type of task was not a significant moderator of the mental simulation effect (see Table 4).

### Dosage: Duration and frequency of mental simulation

To examine the link between dosage of mental simulation and the effect on behavior, we conducted a meta-regression on two components of dosage independently; *duration* and *frequency* of mental simulation training (for a similar approach, see Driskell et al., 1994; Feltz and Landers, 1983). We assessed whether each factor significantly predicted effect sizes.

The mean duration of mental simulation was 23.59 mins (*SD* = 73.02; range: 1–350 mins; median = 3), with the most common being 1 minute (*k* = 36) and the next most common being 3 minutes (*k* = 28), then 5 minutes (*k* = 17). Mental simulation durations were converted into a categorical variable, coded as either *short* (1–5 mins), *medium* (6–20 mins), or *long* (21 mins+).^4^ It was found that effect size was not moderated by the duration of a mental simulation.

To determine frequency of mental simulation, we recorded the amount of mental simulations. The average amount of simulations was 246.08 (*SD*= 1,680.28, range: 1–18,000, median = 3) indicating substantial variability. Meta-regression with total amount of mental simulations established that frequency of mental simulation did not moderate effect size. A final analysis with dosage using total time (Duration × Frequency of Mental Simulation) similarly indicated no moderating effect.

This illustrates that, typically, mental simulation interventions are of a short duration and that simply increasing frequency of simulation does not systematically alter observed effects.

### Delay

It is possible that delay (or time/retention interval) between the last mental simulation and the final measure of behavior accounts for variability in the effect sizes. Although the behavioral test was most frequently administered on the same day as the intervention (*k* = 66, 59% of all 112 comparisons that reported delay), some studies were designed with long delays, with a maximum of 210 days delay (mean delay across studies = 12.61 days, *SD* = 33.96). Nevertheless, it was found that delay did not predict the size of the mental simulation effect (see Table 4).

### Incentive

Of all comparisons, only *k* = 49 (40%) provided enough information to categorize the study as either offering an extrinsic incentive (e.g., course credit, gifts, cash) or offering none. When no incentive was provided, the effect size was not significantly different than zero (*g* = 0.06, *k* = 8) whereas when participants were offered a form of incentive the effect size was 0.37 (*k* = 41) and significantly different than zero. Nonetheless, the difference between this nonsignificant (no incentive) and significant effect is not itself statistically significant (see Table 4).

### Moderation analyses: Overall summary

To briefly summarize the main results of the moderation analyses (displayed in Tables 3 and 4), none of the omnibus tests for the variables tested showed statistically significant effects at the .05 level. Thus, our conclusions are primarily based on identifying and comparing different levels of each variable, based on their Hedges’s *g* and whether each ES was significantly different than zero or not. The exception was our exploratory moderation analyses which showed that purpose (inferior, standard, superior) had a significant moderating effect on effects sizes, and specifically, that standard and superior simulations have more beneficial effects on behavior than inferior simulations.

### Methodological characteristics

Risk of bias was assessed based on five indicators (see Method). Across each of these indicators, the majority of studies (67%–86%) did not provide enough information to classify and meta-analyze. The resulting data were tested as moderators in Table 4.

Studies using a pre–post design in which effect sizes represent pre–post change scores, typically reported more conservative estimates (*g* = 0.46, 95% CI [.163, .747]) than effect sizes incorporating only postintervention scores (*g* = 0.50, 95% CI [.368, .630]), although the difference was not statistically significant (see Table 4). In addition, studies using prescores and postscores showed effects that are also more uncertain and dispersed, as indicated by the wider ranging confidence interval for this effect that approaches zero on the lower limit.

Participant and experimenter blinding did not significantly affect the results. While experiments in which participants were not blinded typically reported much larger effects (*g* = 0.83) than when participants were blinded (*g* = .60), the difference is not significant (see Table 4). Similarly, while studies where experimenters were not blinded (*g* = .72) typically reported (much) larger effects than when experimenters were blinded (*g* = .38), the difference was not statistically significant (see Table 4). These results, however, lack power to detect significant differences (only 29 and 23 experiments respectively) and for those that do give information, nonblinding is rare (only three and nine experiments are nonblinded to participants and experimenters, respectively).

Finally, *allocation* concealment did not affect the results. Studies in which the participants were not aware of which condition they were allocated to typically showed slightly larger effects (*g* = .72) than nonconcealment (*g* = .57) but the difference is not significant (see Table 4). As there are no studies for which we have information that the *outcome* was not concealed, we cannot examine any differences for this methodological characteristic.

### Manipulation checks

As stated in the Introduction, it was important to assess the percentage of studies that measured mental simulation in the control and mental simulation conditions, to verify fidelity to the manipulation. An assessment of all included control–experiment comparisons established that only 16% measured mental imagery in the control condition to assess spontaneously used mental imagery. However, this did not modify the effect size. As seen in Table 4, the effect size was significantly above zero regardless of this manipulation check. Furthermore, approximately half of the experiments (56%) employed active control conditions, whereas the remainder utilized a passive or “wait” control condition (active controls may be more effective at eliminating imagery use in control conditions). Fifty-seven percent of control–experiment comparisons measured mental imagery to confirm basic compliance to the intervention, but, as Table 4 shows, this did not significantly moderate effect size.

## Discussion

In this meta-analysis, our aims were to elucidate for the first time the unique effects of mental simulation on behavior change across a range of domains and uncover which mental simulations, and which intervention characteristics, moderated this effect. In brief, are there identifiable characteristics that enhance the behavioral effect of mental simulation? To answer this question, we improve on previous reviews by (1) conducting a thorough search of all randomized controlled studies on the mental simulation effect and (2) using multilevel modeling to assess the overall effect and potential moderators. The following discussion considers the theoretical and practice implications of the present findings.

We confirmed findings from previous reviews, indicating an overall moderate positive effect of mental simulation compared with nonmental simulation control conditions (Conroy & Hagger, 2018; Driskell et al., 1994; Feltz & Landers, 1983). In an initial meta-analysis, Feltz and Landers (1983) established that mental simulation had a reliable moderate effect (*d* = .48, 60 experimental–control comparisons) on behavior when compared with no practice. Driskell et al.’ (1994) analysis replicated these results with more robust inclusion criteria (*d* = .53, 62 comparisons). In a more recent meta-analysis focusing on health studies by Conroy and Hagger (2018), with 33 comparisons, a similar effect size was found, showing that, on average, mental simulation substantially improves behavior by approximately half of one standard deviation. In the present review, we found a highly similar effect to these reviews (*g* = .49, 123 comparisons) when only including randomized controlled experiments.

The greater power (and multilevel nature) of our analyses, based on over double the number of comparisons of previous reviews, highlights the robustness of this effect (although we should note that actual physical practice has been found to be more effective than simulation; Driskell et al., 1994). That this was consistently demonstrated across such a diverse range of behaviors—from laboratory-based reaction time tasks to slalom skiing and to social behaviors—underlines the reliability and robustness of this effect.^5^ This finding corresponds well with proposals that mental simulations can be adopted as an effective behavior change technique that links thought or intentions with action (Johnson & Sherman, 1990; Pham & Taylor, 1999; Taylor & Pham, 1996) and validates continued professional applications of mental simulation in sports (e.g., Nicholls, 2017) and health (e.g., Hagger, 2016). The size of the effects associated with mental simulation are much larger than those associated with other behavior change techniques, such as self-incentives and self-rewards (*d* = 0.17; Brown et al., 2018). The implication is that mental simulation may be a particularly potent behavior change technique that could be a starting point for the development of more complex behavior change interventions.

The present review has multidisciplinary implications for mental simulation research. Namely, unlike previous reviews, we provide an integrative approach, and identify key classes of mental simulations. To this end, three classes of mental simulations were identified and compared (performance, outcome and process simulations). This integrative approach will enable researchers to assess which types of mental simulations are most effective, and which are relatively inconsequential. It also allows generalities across domains to be interpreted, highlighting underresearched categories of simulation. For example, this meta-analysis showed that process and outcome simulations, but not performance simulations, have been successfully applied in health psychology. This review also highlights the opportunity to develop behavioral interventions across domains where mental simulation is in its infancy (such as social psychology; see Meleady et al., 2013).

The multilevel meta-analytic approach employed here allowed us to examine whether effect sizes were moderated by the subtype of mental simulation. First, we categorized all experimental conditions into one of the categories identified in a taxonomy based on possible subtypes of simulation (see Table 1). Examination of existing studies led to the addition of composite categories (e.g., *process with superior outcomes*), and reduction of others (no *positive process* or *inferior process* simulations were found; although it remains to be seen whether future studies utilize these). All experimental conditions identified were labeled with one of the remaining eight subtypes. Although the planned omnibus test used in the analysis did not reveal a significant effect, we examined whether each level of the categorical moderator leads to either a null *or* statistically significant positive effect, in a one-by-one fashion, replicating a common approach (e.g., Adriaanse et al., 2011; Conroy & Hagger, 2018). Adopting this technique, some subtypes were more effective than others. These differences warrant interpretation and discussion.

### Superior mental simulations improve targeted behavior

One finding was clearly distinguishable and specific: subtypes of mental simulations with positive components (*superior performance*, *superior outcome* and *superior outcome combined with process simulations*) consistently led to reliable positive behavioral effects (see Table 3). This was confirmed by an exploratory multilevel meta-regression analysis of purpose (inferior, standard, superior) showing that superior simulations led to better behavioral outcomes than inferior ones. What mechanisms explain these effects? Here, it is important to apply explanations to each subtype in turn as they represent different ways humans conceptualize the future (see Introduction and Table 1).

The effects of *superior performance* simulations may be understood in terms of the idea that mental and physical practice of a specific behavior are assumed to rely on overlapping neural correlates, dynamic qualities and temporal durations (Allami et al., 2008; Decety & Grèzes, 2006; Jeannerod, 2001; referred to herein as the *overlapping systems hypothesis*). If performance simulations share common neuropsychological processes with actual performance, reasonably, simulating a superior version of that behavior should garner more beneficial changes in behavior than other versions (standard, inferior). Thus, when one imagines optimal performance on a finger-pointing task (Debarnot et al., 2011) subsequent behavior on the task improves, and across studies included here, such simulations led to a large behavioral effect compared with controls (Hedges’s *g* = .67).

The same logic cannot be applied to *superior outcome* simulations, however, because such simulations are, by definition, focused upon the consequences of the enacted behavior rather than the behavior itself (i.e., *if you perform well, you will attain outcome X*, such as feeling positive and physically active; Andersson & Moss, 2011). Benefits of superior outcome simulations are often explained by the motivational “pull” of simulating a desired scenario (Hagger et al., 2011; Vasque & Buehler, 2007). Superior outcome simulations typically focus upon simulating emotions, rather than action plans; they are motivational rather than volitional (Taylor & Pham, 1996). This may be why simply thinking about a desired future is seen as inadequate as a behavior change technique on its own (Pham & Taylor, 1999; Taylor & Pham, 1996). Indeed, if the simulation involves fantasizing, motivation may *decrease* due to the individual feeling a premature sense of achievement in-the-now, reducing the drive to strive for an end goal (Oettingen, 2012). The results of the present meta-analysis are in line with research on possible selves (Markus and Nurius, 1986, b)—that is, simulations of positive scenarios which increase motivation towards that goal, breeding energization and action. Thus, it is apparent here that simulation of superior outcomes (and positive simulations in general) is among several beneficial mental simulation subtypes which can be used as behavior change techniques. However, it is important to state that the effect of superior *outcome* simulations (Hedges’s *g* = .23) was smaller than effects found for other subtypes—possibly because the effect of positive simulations are moderated by factors such as personality type and imagery content (Vasque & Buehler, 2007) or because these simulations are *by nature* less effective.

### No evidence found for an effect of inferior mental simulations on behavior

In contrast, when including composite categories, all subtype incorporating a negative purpose (*inferior outcome*, *inferior performance, process + inferior outcome*) failed to show a significant positive behavioral effect. The difference between inferior and standard/superior simulations was also evident in the moderation analysis of purpose. Thus, in the context of this meta-analysis comparing only experiments on behavioral measures in healthy population, mental simulations incorporating an inferior component (e.g., imagining not withstanding pain: Alden et al., 2001; or gaining weight: Marszał-Wiśniewska & Jarczewska-Gerc, 2016) do not appear to benefit future behavior. With inferior outcome simulations (used separately or combined with process simulations), one might expect a *reduction* in undesirable behaviors (e.g., unhealthy eating)—sometimes called “avoidance behaviors”—by simulating the negative consequences of those behaviors (e.g., weight gain, see Elliot, 2006; Lang, 1995): an ironic effect. Indeed, Taylor’s (1991) examination of the mobilization-minimization hypothesis supports the idea that negatively valenced information, in general, recruits stronger cognitive, social and behavioral responses than does neutral or positive information. However, it is important to clarify that our conceptualization of inferior simulations does not have a one-to-one mapping on to what has been termed negative or negatively valenced simulations (Gamble et al., 2019).

In the context of this meta-analysis, we found that simply imagining *inferior outcomes* was largely inconsequential for behavior. No subtype of simulation with an inferior component garnered reliably positive behavioral effects, whether mapped to the actual performance or the outcomes of behavior. Thus, by including for the first time inferior subtypes of mental simulation in a meta-analysis (when conceptualized differently than emotionally negative simulations; see Gamble, Moreau, et al., 2019), we present novel evidence that disentangles effects that were previously studied homogenously.

It is clear, as anticipated by Pham and Taylor (1999) more than 20 years ago, that more systematic examinations of positive *and* negative outcome simulations (and their potential mediators) are required. Although researchers may want to demonstrate positive behavioral effects, a nuanced understanding of how and why simulations affect behavior (both negatively and positively) requires more complex experimental studies which randomize participants to superior, standard, and inferior simulations within the same study.

The (contrasting) effects of superior and inferior simulations found in this review should also be observed in the context of detailed reviews of mental imagery emphasizing the powerful physiological and cognitive effects of emotional imagery (Decety & Grèzes, 2006). This is because it is likely that superior and inferior simulations are associated with positive and negative emotions, respectively. One area that has examined the effects of negatively emotional simulations is clinical psychology. For example, recent studies have showed an inverse relationship between negative simulations of goals at baseline and goal attainment after a delay of two months (Gamble Tippett, et al., 2019) and that highly negative emotional simulations can predict or maintain clinical symptoms (in depression: Holmes et al., 2016; bipolar disorder: Hales et al., 2011). Nevertheless, we are cautious about overgeneralizing the findings of this meta-analysis to other domains, and stress that the absence of evidence (of an effect of inferior simulations) is not evidence of absence (of an effect). Future research would benefit from determining the independent effects of emotion on effects of these types of simulations.

### Limited and equivocal evidence that mentally simulating the process improves targeted behavior

In the present meta-analysis, when participants focused upon instrumental plans toward one’s goal, rather than goal outcomes—the means rather than the end^6^—there was a small positive effect that did not reach statistical significance within our multilevel moderation model. This does not therefore clearly validate Taylor and colleagues’ (Pham & Taylor, 1999; Taylor et al., 1998) theoretical work on the effectiveness of process simulations for subsequent behavior and self-regulation. The benefits of process simulations are often mentioned (across cognitive and health psychology; Murru & Martin Ginis, 2010; Schacter, 2012) with several authors assuming that such simulations are able to improve one’s decision-making about future acts—helping one decide when, where and how to achieve an end goal. Thus, a positive behavioral effect is implied. However, results from this meta-analysis show that caution is needed in making any strong assumptions about process simulations, at least until further empirical research conclusively replicates positive effects following process simulations. It is noteworthy in this context that the empirical study often cited (e.g., Epstude et al., 2016; Freund & Hennecke, 2015) as key evidence supporting this principle, Pham and Taylor (1999) failed to find a statistically significant effect of process simulation on exam performance (behavior) over the control group (*p* < .09, coded herein as *d* = 0 based on our meta-analytic strategy). Nevertheless, the small amount of studies identified herein (*k* = 5, with only studies conducted since Pham & Taylor, 1999) which fit our criteria and focus on process simulations shows that this research is in its infancy, and we recommend that more empirical research is needed before claims are made about the behavioral benefits of process simulations.

### Mentally simulating performance improves behavior

The present meta-analysis found a significant and reliable effect of standard performance simulation on behavior. Also, an exploratory analysis found that standard simulations worked similarly to superior simulations, and outperformed inferior ones. There are clear, well-documented streams of research that allow explanation of the positive effects of simply imagining a task one will later perform (i.e., *performance simulations*)*.* Traditional explanations of mental simulation’s effects on behavior focused on the idea that humans can mentally replicate a physically practiced/modeled behavior using similar cognitive and neurobiological processes as those necessary for its physical enaction (Corbin, 1972; Feltz & Landers, 1983). In terms of cognition, internalized representations of a specific action purportedly follow similar biomechanical and cognitive rules as actual behavior (Driskell et al., 1994). Furthermore, neuroscientific findings have lent much support to the *overlapping systems hypothesis* (see Decety & Grèzes, 2006; Jeannerod, 2001, for reviews). Applying this principle to the studies included here, through repeated mental practice, one can “engrain” certain behavior chains (or “behavioral schemata”), from initiation to completion, such that behavior chains are retrieved and enacted *automatically* (for example, once a golf putt is repeatedly *simulated*, the same process can be activated and enacted, relatively automatically, when a golfer holds a starting position *in an actual game*; Budney & Woolfolk, 1990).

This mechanism accords well with dual-process theories of behavior which emphasize that behavior is coordinated via two parallel routes; an automatic process, which is rapid and stimulus driven, and a volitional process which relies on conscious intention and reflection (Strack & Deutsch, 2004, see also Hagger et al., 2017).^7^ Under this framework, performance simulations change behavior via a mostly automatic process. In contrast, *outcome simulations* do not involve rehearsal of the to-be-performed behavior, and thus cannot be explained by the instantiation of an automatic process.

Due to their ability to explain a diverse range of psychological phenomena, dual-process models such as the one proposed by Strack and Deutsch (2004) could be useful to distinguish the processes through which *process, performance* and *outcome simulations* lead to changes in behavior (which may utilize reflective and/or impulsive processes, respectively). Applications of this dual-process model to behavior change (such as Hagger et al., 2017), specifically concerning mental simulations, represent a possible fruitful avenue of research revealed by the current meta-analysis and a recent meta-analysis by Conroy and Hagger (2018).

### Combining subtypes of simulations

A sizeable minority of studies combined subtypes of simulation; specifically, here we found several studies that integrated process and outcome simulations. Within this category, the most numerous were mental contrasting interventions—a technique devised by Oettingen and colleagues (Oettingen, 2012) to combine volitional and motivational aspects of mental simulation (typically aligned with *process* and *outcome simulations*, respectively, yet distinct from this conceptualization). There exists supportive evidence spanning over 15 years showing positive behavioral effects of mental contrasting (e.g., Oettingen & Mayer, 2002; Oettingen & Wadden, 1991; see Oettingen, 2012, for a review). Our data largely agreed. Although not the largest, a homogenous positive effect was found when examining combined process *and* superior outcome simulations. On the other hand, only two studies (both indicating null effects) were identified incorporating *process and inferior outcome* simulations. Thus, here we did not have enough data to draw firm conclusions.

### Summary of effects as a function of subtypes of mental simulation

In summary, although the present meta-analysis replicates similar positive effects as found previously (e.g., Driskell et al., 1994), we show that this effect depends on different classes of mental simulation. Furthermore, these different classes could rely on different mechanisms. This is important for theoretical developments and practical applications of this technique because specific classes of mental simulation could be more appropriate for specific situations and populations (e.g., especially clinical populations with cognitive or emotion dysfunctions).

However, there are limits to what these data say about “standard” mental simulations. Fundamentally, because our types of mental simulation were based on differences in instruction, this opens the question of what kinds of simulation participants engaged in when not directed to imagine either a superior or inferior behavior (“standard” simulations; see Table 1). Although we assumed these simulations would fall “in-between” inferior and superior simulations, it is possible that when undirected, participants imagine idealized scenarios (i.e., “future positivity bias”; see Berntsen & Bohn, 2010). It would be important for future research to confirm whether such a systematic positivity bias exists in the types of mental simulation employed in studies included in this meta-analysis.

### Do incentives moderate the mental simulation effect?

We found a significant moderating effect for incentive, a variable selected for analysis for the first time based on a related theoretical framework (Michie et al., 2013). Extrinsic incentives (i.e., vouchers, money, course credit) influenced the effectiveness of mental simulation interventions such that mental simulations without incentives failed to produce a significant effect (*g* = .06 on average), whereas those including rewards garnered significant positive effects. Although based on a small sample of studies (some with small *N* sizes) this represents a novel avenue to examine in the mental simulation literature and supports our hypothesis that offering rewards represents a behavior change technique based on increased motivation (Brase, 2009), and would “boost” any effects of mental simulation. Importantly, this boost was not the result of contingencies within studies whereby the reward was tied to the mental simulation. Indeed, in all relevant studies, incentives were given independently of group assignment. This finding raises the possibility that public health programs utilizing mental simulation interventions should strongly consider combining them with extrinsic reward and indicates the that primary research in this area would be fruitful.

### Other methodological characteristics

For the remaining moderating variables (*dependent variable*, *domain*, *dosage*, *delay, task type*, *incentive,* and *study quality*), no moderating effects were found. It was somewhat surprising that increased frequency and duration of mental simulation show no impact on behavior (although see Driskell et al., 1994, for a similar finding). It is probable that a minimal dosage is required for interventions to be cost and time effective (the “optimal duration” was found to be 20 minutes in Driskell et al., 1994), after which successive simulations do not garner additional benefits. We recommend that future empirical work directly tests the effect of dosage. Specifically, well-controlled experiments manipulating the temporal spacing (e.g., massed versus distributed) of mental simulation sessions while holding amount of dosage constant will be required. One testable hypothesis would be that massed practice leads to less effective outcomes than distributed practice (Corbin, 1972)—a robust finding recently shown in studies of learning (see Dunlosky et al., 2013, for a review).

There was also no evidence for a reduction of the mental simulation effect over time, consistent with Conroy and Hagger (2018; this conflicts with Driskell et al., 1994). The lack of an effect here may be due to the fact that our delay effect was based on the time lag between last mental simulation and *farthest* time point measured to avoid overestimating the mental simulation effect. In Driskell et al. (1994), it was unclear how delay was measured and whether intermediate or “booster” procedures were incorporated. Thus, here we show when the final impact of the intervention is considered, the positive effects of mental simulations are not limited by the delay.

### Limitations

Two main limitations of this meta-analysis require elaboration. First, although we assessed subtype differences in a one-by-one fashion, the reason why these apparently substantial differences in effect sizes (see Table 3) did not lead to a significant moderation effect on the omnibus test should be considered. We note that this null effect could be due to low power to detect large effects due to small *k* sizes per subtype and small sample sizes within the included studies. This meta-analysis also included studies using different tasks, outcome measures, and domains. Although this allowed us to analyze which effects stand across experimental variation, it also increases the chance that some real effects, specific to certain tasks, outcomes and domains, were masked by general methodological variability (a common issue in meta-analyses; Lipsey & Wilson, 2001).

Secondly, we restricted our search to peer–reviewed publications. The exclusion of unpublished work and the gray literature (e.g., conference proceedings) that are sometimes included in meta-analyses (e.g., Conroy & Hagger, 2018; Harkin et al., 2016), probably did not affect the validity of our findings: We did not find evidence of publication bias (i.e., there was no relation between low precision and effect size in the studies included; see Fig. 2).

### Future directions

In future research, it will be important to expand the present analysis of mental simulation subtypes, which was restricted here due to the commonality of some interventions (e.g., performance simulations) and the scarcity (e.g., negative process simulations) of others. Indeed, three of the nine cells identified in possible subtypes of mental simulation (see Table 1) could not be examined due to lack of empirical research. This sets the scene for new research to examine untested subtypes of simulation, such as inferior or superior process/plans versus an appropriate control.

Another fruitful avenue for future work would be exploring the longitudinal effects of mental simulation. The majority of studies included in this review assessed behavioral effects within 1 day of the intervention, and none examined effects surpassing one year. Similarly, the finding that almost all studies are conducted with young student participants drives the need for more mental simulation studies among diverse and more inaccessible populations.

Furthermore, our analysis of domain indicated the need for studies to examine the effect of “open” and “closed” behaviors. For example, one study included here examined this factor within badminton (serving = closed; returning = open; Jeon et al., 2014), defining open skills as “generally more variable, unpredictable and externally paced,” whereas closed skills are “more predictable, stable and self-paced” (p. 157). It is possible that some mental simulations (e.g., performance simulations) might be less effective for open skills, being less able to map directly onto the future behavior, and draw upon the overlapping systems underlying behavior and mental imagery of behavior (Decety & Grèzes, 2006). Similarly, in health psychology, researchers may require interventions that advocate flexible, open behaviors (e.g., in selecting locations to eat healthily), driving a need to consider whether the type of simulation utilized for an intervention lends itself to “open” or “closed” behaviors.

Moreover, the difference between open and closed behaviors links with a more conceptual issue: whether some of the simulations can be defined as future oriented or if some are merely reactivated memories. We outline two types of simulation that may or may not be reactivations of memory. Firstly, where a participant is asked to simulate a circumscribed fixed pattern of behavior, they may indeed rely on a memory activation when simulating the task (e.g., a “closed” behavior; see above). This would involve no “temporal tag” as a future event. Secondly, where a participant must put him or herself into a future context or where behaviors are more flexible (e.g., “open” tasks), an explicit simulated event is required with a future “temporal tag.” Research has shown that such “episodic future thoughts” (Atance & O’Neill, 2001), although relying on memory processes and brain structures, involve a constructive recombination of episodic details into a novel simulated scenario (see Schacter et al., 2012, for a review). Current models of mental simulations do not fully account for these differences, highlighting the need for further theoretical work.

This meta-analysis highlighted a need for studies to systematically include a manipulation check to assess whether participants in experimental conditions actually created a mental image in their mind’s eye—whether that be a verbal description of the image (Hagger et al., 2011), a rating of vivid imagery (Andre & Means, 1986), or a judgment of imagery duration (Debarnot et al., 2011) related to the simulation. Furthermore, the moderation analyses herein rest somewhat on the assumption that participants were indeed simulating in the way they were instructed. Although there was no strong evidence to the contrary (i.e., that participants were regularly excluded for simulating different scenarios/behaviors), and over half of the included studies did carry out a manipulation check on simulation content, it would be important for reliability that all future studies verify the content of the simulation with such a manipulation check. Importantly, very few studies checked whether those in the control condition engaged in mental simulation (in which opportunities exist to simulate the subsequent behavior), indicating a need for studies to include a retrospective check. Although a moderation effect of verification was not found, if a check does not happen, the mental simulation effect may be reduced due to imagery use across experimental and control conditions.

Finally, according to a review of placebo effects (Boot et al., 2013), an adequate control condition in psychological interventions should ensure that control participants’ expectations about potential benefits of the “intervention” should match those in experimental conditions. Although approximately half of all studies included here adopted active controls (e.g., reading poetry), it is likely that those in the control and experimental conditions differed on whether and how much they expected positive behavioral effects from “interventions” they received. For instance, those in true experimental conditions may realize similarities between the simulation and task, and guess they are in the experimental arm; those in control conditions may, in contrast, realize the “irrelevant” nature of the control “intervention.” This would affect expectations of task success. This limitation of previous studies can be overcome by measuring outcome expectations after intervention/control (Boot et al., 2013), or by informing those in the experimental group they are in the control group, and assessing feedforward effects on behavior.^8^

## General summary

Several authors have argued and built models around the proposal that mental simulation is a desirable method to increase optimal behaviors (e.g., Oettingen, 2012; Taylor & Pham, 1996). Here, we analyzed if those benefits extended across several conditions, as defined by a taxonomy informed by extant empirical research. We found that across a range of behaviors, classifications identified provided a useful framework to differentiate subtypes of simulations and their effects. These findings not only have clear implications for theoretical understanding of mental simulation effects but may aid professionals seeking evidence-based and cost-effective methods of changing behavior. It is hoped that a common language will motivate more cross-pollination across subdisciplines. Furthermore, new research programs will benefit from delineating not only *in which circumstances* but also *how* mental simulation changes behavior (see Michie et al., 2013). These new research programs may benefit from a variety of methodological approaches: incorporating individual differences, experimental, and neuroscientific approaches. Finally, important insights may be gained by building upon effective applications of mental simulation in the domains of psychotherapy and neuropsychology (Holmes et al., 2007; Liu et al., 2004).

## Supplementary Information

ESM 1 (DOCX 71 kb)

ESM 2 (CSV 21.8 kb)

## Footnotes

1 One type of intervention relevant here is that of mental contrasting (see Oettingen, 2012, for a comprehensive review). This future-thinking intervention involves individuals generating a positive fantasy and then contrasting them with the present barriers to its realization. In doing so, effective plans are devised (so long as certain preconditions are met, such as positive expectations of success; see Oettingen, 2012). The present meta-analysis had an interdisciplinary focus, and thus we were not able to examine possible mediators and moderators (e.g., expectations, attitudes) from specific domains (health and social psychology). Nevertheless, it was expected that several studies utilizing mental contrasting were necessarily included in this review, and could be categorized within the aforementioned taxonomy, if they fulfilled the inclusion criteria (e.g., behavior as the outcome variable). It will be valuable to assess how this taxonomy maps onto other mental simulation interventions used within the current literature.

2 However, it is notable that the majority of control/mental simulation comparisons had only one type of outcome measure (96%), with five having two outcome measures.

3 The hypothesis that the degree of mental imagery would mediate the mental simulation effect has been proposed previously (Lang, 1995; Richardson, 1967). As an exploratory measure, we aimed to identify whether mental imagery capacity (as an individual difference measure) mediated the relation between mental simulation intervention and the behavioral outcome. However, after inspecting each study for quantitative data, only six comparisons reported the relation between mental imagery and behavior. It was therefore unfeasible to carry out meta-analyses of this data (similar to Conroy & Hagger, 2018), so we briefly summarize their results thus: Of the eight that assessed the relation between mental imagery and behavior in simulation conditions, only one reported a significant correlation—indicating a positive association between level of mental imagery and behavior (Spearman’s rho = .62, *p* < .01; Arora et al., 2011). All other studies reported no significant relation between mental imagery and behavior (all *p*s > .77; Callow et al., 2013, Exp. 1 & Exp. 3; Epstein, 1980, Exp. 1; Hayter et al., 2013; Ruffino et al., 2017; Woolfolk et al., 1985).

4 Duration was coded as opposed to using the continuous data of minutes of each simulation, because there were some extremely long durations (e.g., Pascual-Leone et al., 1995), that meant the data would be dramatically skewed. For the sake of completeness, when minutes of each mental simulation was used, the same result was found: That duration of mental simulation did not significantly moderate effect size.

5 Note that the overall effect size is a conservative estimate of the mental simulation effect, as only RCTs were included and analytical measures were taken to reduce overestimation of the average effect size (see Methods: Meta-Analytic Strategy section).

6 However, note that Taylor and colleagues’ comparisons between process and outcome were based on positive outcomes, whereas we additionally compare positive and negative outcomes.

7 Although we adopt a neuro-cognitive explanation here, it is important to note the dearth of social/health research utilizing *performance simulations* (see Supplementary Materials). Hence, it is possible that explanatory variables typically adopted in health/social psychology (e.g., *self-efficacy*, Bandura, 1997; *framing* and *self-appraisal*, see Baumeister et al., 2011) may play a - hitherto undiscovered - role in explaining performance simulations.

8 We thank Mike Page for making the latter point.
