# Supplementary material for: Synthesizing the effects of mental simulation on behavior change: Systematic review and multilevel meta-analysis
Source: Psychon Bull Rev. 2021 May 4;28(5):1514–37. doi: 10.3758/s13423-021-01880-6 (PMC8500882; doi:10.3758/s13423-021-01880-6)
Supplement: Supplementary file 1 — (DOCX 71 kb) [file 13423_2021_1880_MOESM1_ESM.docx]

**Table 2:** Characteristics of Included Studies, Including Potential Moderators

| **Study** | ***N*** | **Age** | **Sex %**  ♀ | **Sample + ethnicity** | **Incentive** | **Outcome** | **MS duration (mins)** | **MS frequency** | **Delay**  (days) | **Behavior** | **Domain** |
| --- | --- | --- | --- | --- | --- | --- | --- | --- | --- | --- | --- |
| Adams_2014_ Exp1 | C=20, MS^c^=25 | NR | NR | University students | Course credit/$10 | Speed | 1 | Once | 0 | Manual rotation of shapes | Motor skills |
| Adriaanse_2010_  Exp 2 | C =20, MS^h^ =19 | 19.4 YA | 100 | University students | $10 | Frequency | 3 | X 7 | 7 | Eating habits (self-report) | Health |
| Alden_2001 | C=10, MS^d^=10, MS^b^=10 | YA | 50 | University students | NR | Speed | 5 | Once | 0 | Pain tolerance of cold pressor | Pain |
| Allami_2008 | C=5, MS^c^=5 | 28.5  YA | NR | NR | NR | Speed | 1 | X 120 | 0 | Visuo-motor sequence task | Motor skills |
| Andersson_2011 | C = 15, MS^f^ = 15 | 29 YA | 68 | White European university students and employees | None | Frequency | 5 | X 14 | 14 | Physical activity | Health |
| Andre_1986 | C=22,  MS^c^=44 | 20.97 YA | 0 | University students | Course credit | Frequency | 15 | X 5 | 5 | Frisbee-throwing | Sports |
| Arora_2011 | C=9, MS^c^=9 | 22 YA | 33 | Surgeons | NR | Frequency | 30 | X 5 | 0 | Surgical skill | Occupational |
| Austenfield_2006 | C=21,  MS^f^=21 | 26.4 YA | 45 | Students: white (84%),  with 2% African American, 2% Latino, 11% Asian, and 2% other ethnicities | $25-$100 | Frequency | 25 | X 3 | 90 | Medical visits (before and after intervention in 3 month window); fewer the better | Health |
| Baker_1983 | C=14, MS^c^=13 | YA | NR | Counselling students | NR | Frequency | 150 | Once | 7 | Counsellor Interview Competence Scale; Responding Skills Proficiency Index; Attending Skills Proficiency Index | Occupational |
| Budney_1990_ Exp1 | C=10, MS^d^=10;MS^b^=10 | NR | NR | NR | NR | Frequency | 1 | Once | 0 | Golf putting | Motor skills |
| Callow_2013_  Exp1 | C=15, MS^d^=15 | 21.5 YA | 0 | Drivers | NR | Speed | 1 | Once | 0 | Driving in a simulator | Motor skills |
| Callow_2013_  Exp3 | C=10, MS^c^=10 | 24.79 YA | 23 | Recreational skiers | NR | Speed & Frequency | 1 | 10 | 30 | Skiing on an artificial slope | Sports |
| Callow_2017 | C=15  MS^c^=30 | 21.75 YA | 0 | University students | NR | Speed | 2 | X 1 | 0 | Driving on a simulated rally driving circuit | Sports |
| Conroy_2015 | C=60, MS^a^=44, MS^f^=62,MS^h^=45 | 20.16 YA | 74 | University students | £25 prize draw | Frequency | 3 | X 3 | 28 | Weekly Alcohol consumption in Units of alcohol | Health |
| Creelman_2003 | C=13, MS^c^=11 | 28.8 | 43 | NR | NR | Accuracy | 3 | X 2 | 0 | Big toe abduction | Motor skills |
| Debarnot_2011_  Exp1 | C=8, MS^d^=8 | 28.0 | 54 | NR | NR | Speed & Frequency | 1 | X 10 | 0 | Finger tapping sequence | Motor skills |
| Epstein_1980 | C=15, MS^c^= 30 | YA | 44 | University students | NR | Frequency | 3 | Once | 0 | Dart-throwing | Sports |
| Fery2003_Exp1 | C=8, MS^d^=8, | 21.9 YA | 0 | University students | NR | Frequency | 1 | X 36 | 2 | Reproduction of an image (by drawing) | Motor skills |
| Fery2003_Exp2 | C=8, MS^d^=8 | 20.5 YA | 0 | University students | NR | Frequency | 1 | X 12 | 2 | Reproduction of an image (using a stylus) | Motor skills |
| Fontani_2007 | C=10,  MS^c^=10 | 35 | 0 | Karate students | NR | Speed & Frequency | 1 | X 3600 | 1 | Power and strength in karate | Sports |
| Gentilli_2010_ Exp1 | C=10,  MS^d^=10 | 20-24 YA | 50 | NR | NR | Speed | 1 | X 60 | 0 | Pointing task | Motor skills |
| Gentilli_2010_ Exp2 | C=5,  MS^d^=5 | 24.3 YA | NR | NR | NR | Speed | 1 | X 60 | 1 | Pointing task | Motor skills. |
| Geoffrion_2012 | C=26, MS^c^=24 | Not reported | 74 | Novice surgeons | NR | Frequency | 3 | X 3 | 1 | Medical operation | Occupational |
| Gomes_2014 | C=12, MS^d^ =24 | 24.7 YA | Mixed | University students | NR | Frequency | 3 | X 6 | NR | Physical sequencing task (error) | Motor skills |
| Gould_1980_Exp2 | C=20; MS^d^=20 | Not reported | 50 | University students | NR | Frequency | 1 | X 4 | 0 | Strength | Motor skills |
| Grouios_1992 | C=20; MS^c^=20 | 18-25 YA | 0 | Competitive sports people | NR | Speed | 1 | X 560 | NR | Reaction time task | Motor skills |
| Hafenbräld_2018_Exp2 | C=51;  MS^c^=50 | 36.0 | 56 | MTurk community sample | Up to 1.20 USD | Accuracy | 3 | X 1 | 0 | Simulated auction task | Social |
| Hagger_2011 | C=73; MS^f^=86 | 35.7 | 39 | Corporate employees with varied SES backgrounds and positions | Prize draw | Frequency | 3 | Once | 28 | Units alcohol consumed, binge drinking episodes (self-report) | Health |
| Hagger_2012a | C=168, MS^f^=181 | 20.2 YA | 74 | Students (from Estonia, UK and Finland) | Prize draw (£100) | Frequency | 3 | Once | 28 | Units alcohol consumed, binge drinking episodes (self-report) | Health |
| Hagger_2012b | C=269, MS^f^=169 | 22.8 YA | 56 | University students | Prize draw | Frequency | 3 | Once | 28 | Units alcohol consumed, binge drinking episodes (self-report) | Health |
| Halvari_1996 | C=23, MS^c^=22 | YA | 42 | Sports class students | NR | Speed & Frequency | 13 | X 3 | 0 | Sequence of physical exercises | Sports |
| Hayter_2013 | C=21, MS^c^=19 | Not reported | 40 | Postgraduate anaesthesia trainees | NR | Frequency | 20 | Once | 0 | Medical Crisis Management (Ottowa Global Rating Scale) | Occupational |
| Jeon2014_Exp1 | C=12; MS^c^=12 | 23.3 YA | 0 | University Students | NR | Frequency | 1 | X 3 | 0 | Serving in badminton (closed skill) Error | Sports |
| Jeon2014_Exp2 | C=12; MS^c^=12 | 21.1 | 50 | University Students | NR | Frequency | 1 | X 3 | 0 | Returning in badminton (open skill) Error | Sports |
| Johannessen_2012 | C=30; MS^h^=36; MS^f^=35 | 19.6 | 92 | University Students | Course credit | Frequency | 3 | Once | 14 | Calorie intake, high calorie foods (self-report) | Health |
| Jungmann_2011 | C=20; MS^c^=20 | Not reported | 50 | Medical students | NR | speed | 3 | X 4 | Variable (not used in meta-analysis) | Virtual reality surgical performance | Occupational |
| Kirk_2013 | C=23; MS^h^=22 | Not reported | 45 | University Students | Course credit | Frequency | 3 | Once | 0 | Effective negotiation (joint gain) between pairs of individuals | Social |
| Knauper_2011 | C=44; MS^f^=34 MS^a^=37 | 18.3 | 62 | Students. 71.8% ‘White’, 10.7% ‘Asian’, 6.2% South Asian, 2.8% Hispanic, 8.5% as ‘other’. | NR | Frequency | 5 | X 7 | 7 | Fruit intake (self-report) | Health |
| Kohl_1980_Exp1 | C=21; MS^c^=19 | 21.0 | 0 | University Students | NR | Frequency | 1 | X 25 | 0 | Pursuit Rotor task performance (opposite hand to mental simulation) | Motor skills |
| Kohl_1980_Exp2 | C=20; MS^c^=20 | 20.3 | 0 | University Students | NR | Frequency | 1 | X 18 | 0 | Pursuit Rotor task performance (opposite hand to mental simulation) | Motor skills |
| Kohl_1980_Exp3 | C=36; MS^c^=36 | 16.9 YA | 0 | High school students | NR | Frequency | 1 | X 9 | 0 | Pursuit Rotor task performance (opposite hand to mental simulation) | Motor skills |
| Komesu_2009 | C=35; MS^c^=33 | 29.0 | 81 | gynaecology residents | NR | Frequency | 20 | Once | 0 | Cystoscopy | Occupational |
| Kornspan_2004 | C=10; MS^d^=10 | 19.6 | 22 | University students | NR | Frequency | 1 | X 20 | NR | Golf-putting task | Sports |
| LaCourse_2004 | C=12; MS^c^=12 | 23.3 | 54 | University students | NR | Frequency | 15 | X 5 | NR | Pointing task | Motor skills. |
| Lohasz_1997 | C=15; MS^c^=15 | 19-26 | 0 | University athletes | NR | Speed | 1 | X 3 | 0 | Pointing task | Motor skills |
| Lukazewski_2012 Exp1 | C=10; MS^c^ =10, MS^f^ = 10, MS^g^ =10 | 19-26 | 50 | University students | NR | Speed | 5 | Once | 0 | Persistence | Motor skills |
| Lukazewski_2012 Exp2 | C=32; MS^b^=32; MS^d^=32 | 20.4 | 53 | “different educational backgrounds”(p.34) | NR | Speed | NR | Once | 0 | Persistence | Motor skills. |
| Lukazewski_2012 Exp4 | C=30; MS^d^=30 | 26.0 | 52 | “different educational backgrounds”(p.37) | NR | Speed | NR | Once | 0 | Persistence | Motor skills |
| Maring_1990 | C=13; MS^c^=13 | 30.0 | 81 | Students and employees | NR | Frequency | 2 | X 5 | 0 | Throwing task | Motor skills |
| Marszał-Wiśniewska_2016 Exp2 | C=18; MS^h^ = 18; MS^a^ = 18; MS^f^=17; MS^g^=18; MS^e^ = 17 | M=29 (SD=19-45) | 100 | Public visiting a dieting internet forum | No incentive provided (except potential weight loss) | Frequency | 350 | X 35 | 36 | Frequency of log ins to an online weight management programme | Health |
| McNeil_2019 | C=16;  MS^d^=16 | 21.5 | 100 | Athletes | NR | Speed | 20 | X 6 | 1 | Reactive agility test | Motor Skills |
| Meleady_2013 Exp1 | C=27; MS^d^=27 | 19.9 | 75 | University Students | Stationary based on task performance | Accuracy | 5 | Once | 0 | Cooperation in prisoner's dilemma game | Social |
| Meleady2013 Exp2 | C=24; MS^d^=24 | 20.9 | 100 | University students | £3, one group received performance-based pay | Frequency | 5 | Once | 0 | Cooperation in prisoner's dilemma game | Social |
| Meleady2013 Exp3 | C=27; MS^d^=27 | 18.5 | 89 | Students (6^th^ form and university) | one group received performance-based pay | Frequency | 5 | Once | 0 | Cooperation in prisoner's dilemma game | Social |
| Meleady2013 Exp4 | C=17; MS^d^=34 | 20.7 | 84 | University students | Course credit | Frequency | 5 | Once | 0 | Cooperation in prisoner's dilemma game | Social |
| Mendoza1978 | C=8; MS^d^=8 | 19-22 | 19 | University students | NR | Frequency | 15 | X 6 | 1 | Dart-throwing | Sports |
| Meslot2016 Exp1 | C*=30*  MS^a^*=21* | M = 25.1 | 86 | Undergraduate students | NR | Frequency | NR | Once | 28 | Physical activity | Health |
| Meslot2016 Exp2 | C*=18*  MS^a^*=20* | Mean age = 27.44 years | 72 | Fitness centre members | NR | Frequency | NR | Once | 133 | Fitness center attendance | Health |
| Morin2000 | C=10; MS^d^=10 | 46 | 7 | Paper mill employees | NR | Frequency | 30 | X 4 | 30 | Communication skills | Social |
| Murru2010 | C=15; MS^f^=17; MS^e^=20 | 21.4 | 76 | Students. White-Caucasian  (48%), Asian, (20%), and South Asian (19%) | $5 | Frequency | 3 | Once | 56 | Duration of physical exercise (self-report, diary) | Health |
| O 2008 | C = 24; MS = 72^c^ | 18.1 YA | 68 | University student football players | NR | Speed | 1 | X 3 | 0 | Dribbling a football | Sports |
| Overdorf2004 | C=6; MS^d^=6 | 29.6 | NR | University students | NR | Frequency | 3 | X 36 | 0 | Errors on a reaction time task | Motor skills |
| Pascual-Leone1995 Exp2 | C=9; MS^c^=9 | 32.0 | 40 | NR | NR | Speed | 120 | X 5 | 0 | Piano playing (also error) | Motor skills |
| Peluso2005 | C=30; MS^d^=15 | 18-25 | 73 | University students | Course credit | Frequency | 1 | X 10 | 0 | Golf putting performance | Sports |
| Pham1999 | C=21; MS^f^=21; MS^a^=21; MS^h^=21 | 18-28 | 73 | University students. 29.7% Anglo, 44.6% Asian, 10.9% Latino,  5.9% African American, and 8.9% other. | Course credit | Frequency | 5 | X 6 | 1 | Examination performance | Occupational |
| Post_2018 | C=14;  MS^d^=14 | 20.6 YA | 100 | University students | NR | Speed | 1 | X 54 | 1 | Reaction time task | Motor skills |
| Raison_2018 | C=17;  MS^d^=20 | 23.55  YA | 40 | Junior doctors/ medical students | NR | Frequency | 30 | X 1 | 0 | Simulated surgical skills | Occupational |
| Rawlings1972 Exp1 | C=8; MS^c^=8 | NR | 100 | University students | NR | Frequency | 1 | X 8 | 1 | Rotary pursuit task | Motor skills |
| Rawlings1972 Exp2 | C=10; MS^c^=10 | NR | 0 | University students | NR | Frequency | 13 | X 8 | 1 | Rotary pursuit task | Motor skills |
| Renner_2019 | C=48;  MS^d^=24 | 36.35 | 68 | Community volunteer panel | Monetary payment | Frequency | 12 | X 15 | 0 | Completing daily activities | Health |
| Ross_1985 | C=6; MS^c^=6 | 22.0 | 3 | Music students specializing in trombone | NR | Frequency | NR | X 3 | 0 | Trombone playing | Motor skills |
| Rozand_2016 | C *=* 19*;* MS^d^ = 12 | M=24.3, SD=2.5 | 45 | NR | NR | Speed | 1 | X 100 | 0 | Pointing task | Motor skills |
| Ruffino_2017 | C = 10;  MS^d^ =10 | M = 27 | 50 | NR | NR | Speed | 1 | X 30 | 0 | Nine Hole Peg Test | Motor Skills |
| Ryan_1986 | C=20; MS^c^=20 | NR | 72 | University students | NR | Frequency | 1 | X24 | 0 | Throwing task | Motor skills |
| Saimpont_2013 | C=10; MS^c^=10 | 27.4 | NR | NR | NR | Speed | 1 | X 720 | 206 days | Motor sequence with left foot | Motor skills. |
| Sanders_2008 | C=31; MS^c^=31 | NR | NR | Medical students | NR | Frequency | 30 | X 2 | 10 | Surgical procedure | Occupational |
| Seif-Barghi2012 | C=17; MS^d^=17 | 19.4 (13-32) | 0 | Elite football players; four age categories, U19, U21, over 21 | NR | Frequency | 15 | X 7 | 0 | Football passing performance | Sports. |
| Shackell2007 | C=10,  MS^d^=10 | 20.1 | 0 | Student football, basketball and rugby players | One protein bar per 15 min session | Frequency | 15 | X 15 | 2 | Strength (hip flexor task) | Motor skills |
| Shanks2000 | C=10; MS^d^ = 10 | NR | 52 | University students | NR | Speed | 7 | Once | 0 | Sequential reaction time task | Motor skills |
| Sheeran2013 | C=42; MS^h^=42 | 53.9 | 0 | Angling club members. Mostly working class 69%  employed; 29.80% retired | Prize draw of annual membership (2 available) | Frequency | 3 | Once | 210 | Physical activity (self-report) | Health |
| Smith2003 | C=6; MS^d^=6 | 29.3 | 0 | Staff and postgraduate | NR | Frequency | 3 | X 8 | 2 | Strength (finger) | Motor skills |
| Smyth1975 Exp1 | C=10; MS^c^=10 | NR | 41 | University Students | No payment | Speed & Frequency | NR | X 5 | 0 | Mirror-drawing task | Motor skills |
| Smyth1975 Exp2 | C=10; MS^c^=10 | NR | 44 | University Students | No payment | Frequency | 4 | Once | 0 | Pursuit rotor task | Motor skills |
| Strachan 2017 | C = 86; MS^f^ = 81; MS^h^ = 77 | 29.6 | 82 | Community sample | $10 gift token | Frequency | 5 | Once | 240 | Physical activity (self-report) | Health |
| Theeuwes_2018_Exp2 | C = 20;  MS^c^ = 20 | NR | NR | University students | 10 euros cash | Speed & Accuracy | 1 | X 15 | 0 | Typing task | Motor skills |
| Vogt1995 Exp1 | C=16; MS^c^=16 | 20-39 | 50 | University Students | 25 DM, extra 25 DM for top performer | Frequency | 1 | X 24 | 24 | Motor task | Motor skills |
| Vogt1995 Exp2 | C=16; MS^c^=16 | 18-36 | 50 | University Students | 20 DM | Frequency | 1 | X 60 | 0 | Motor task | Motor skills |
| Wohldmann2007 Exp1 | C=20; MS^c^=20 | NR | NR | University students | Course credit | Speed | 1 | X 320 | 0 | Typing number strings | Motor skills |
| Wohldmann2007 Exp2 | C=27; MS^c^=27 | NR | NR | University students | Course credit | Speed | 1 | X 640 | 90 | Typing number strings | Motor skills |
| Wollman1985 Exp1 | C=7; MS^c^=7 | NR | 0 | University cross-country track team | NR | Speed | NR | NR | NR | Running | Sports |
| Woolfolk1985 | C = 8; MS^b^; MS^c^; MS^d^; MS^e^; MS^e^, (n=8 in each group) | NR | 0 | University students | Course credit | Frequency | 7 | X 4 | 0 | Golf putting performance | Sports. |
| Yue1992 | C=10;  MS^d^ = 10 | 21-29 | NR | NR | NR | Frequency | 1 | X 300 | NR | Finger strength on a laboratory task | Motor skills |
| Zecker1982 | C=10; MS^c^ = 10 | NR | 50 | University students | Course credit | Accuracy | 1 | X 40 | 0 | Throwing to target | Motor skills |
| Zinatelli1990 | C=10; MS^c^=10; MS^d^=10 | 19-25 | 0 | University students | Cash ($5 each) | Speed | 3 | X 8 | 0 | Pursuit Rotor Task | Motor skills. |

Note: MS duration = The duration of one trial of mental simulation practice. Delay: Any delay within the same day = 0, 1 = 1 day delay and so on; C=Control Group; MS = mental simulation / experimental group; a = process; b = negative performance; c = neutral performance; d = positive performance; e = negative outcome; f = positive outcome; g = negative outcome + process; h = positive outcome + process.; Sex (% females / ♀). PP = physical practice. Delay (Immediate ≤ 5 mins). Duration: To give a conservative estimate of MS duration, where low and upper bounds were provided , the maximum was used for analysis. DM = Deutschmark. Similarly, where numbers were not integers we rounded up, and ‘a few seconds’ was assigned 1 minute. ‘A few’ was assigned 3 minutes in MS duration and MS frequency analyses. In contrast to other studies, in Lukazewski2012 Exp2 participants had no physical practice/demonstration of the task in question before imagining.

Reference

Adams, D. M., Stull, A. T., & Hegarty, M. (2014). Effects of mental and manual rotation training on mental and manual rotation performance. Spatial Cognition and Computation, 14(3), 169–198. https://doi.org/10.1080/13875868.2014.913050

Austenfeld, J. L., Paolo, A. M., & Stanton, A. L. (2006). Effects of writing about emotions versus goals on psychological and physical health among third-year medical students. Journal of Personality, 74(1), 267–286. https://doi.org/10.1111/j.1467-6494.2005.00375.x

Callow, N., Jiang, D., Roberts, R., & Edwards, M. G. (2017). Kinesthetic imagery provides additive benefits to internal visual imagery on slalom task performance. Journal of Sport and Exercise Psychology, 39 (1), 81–86. https://doi.org/10.1123/jsep.2016-0168

Creelman, J. (2003). Influence of mental practice on development of voluntary control of a novel motor acquisition task. Perceptual and Motor Skills, 97, 319–337. https://doi.org/10.2466/pms.2003.97.1.319

Fery, Y. (2003). Differentiating visual and kinaesthetic imagery in mental practice. Canadian Journal of Experimental Psychology, 57(1), 1–10. https://doi.org/10.1037/h0087408

Fontani, R., Facchini, A., Casini, M., & Corradeschi, F. (2007). Effect of mental imagery on the development of skilled motor actions. Perceptual and Motor Skills 105, 803–826. https://doi.org/10.2466/pms.105.3.803-826

Geoffrion, R., Gebhart, J., Dooley, Y., Bent, A., Dandolu, V., Meeks, R., Baker, K., Tang, S., Ross, S., & Robert, M. (2012). The mind’s scalpel in surgical education: a randomised controlled trial of mental imagery. BJOG: An International Journal of Obstetrics & Gynaecology, 119 (9), 1040–1048. https://doi.org/10.1111/j.1471-0528.2012.03398.x

Gomes, T. V., Ugrinowitsch, H., Marinho, N., Shea, J. B., Raisbeck, L. D., & Benda, R. N. (2014). Effects of mental practice in novice learners in a serial positioning skill acquisition. Perceptual and Motor Skills, 119 (2), 397–414. https://doi.org/10.2466/23.PMS.119c20z4

Grouios, G. (1992). On the reduction of reaction time with mental practice. International Journal of Sport Behavior, 15 (2), 141–157.

Hafenbrädl, S., & Woike, J. K. (2018). Competitive escalation and interventions. Journal of Behavioral Decision Making, 31 (5), 695–714. https://doi.org/10.1002/bdm.2084

Hagger, M. S., Lonsdale, A. Koka, A., Hein, V., Pasi, H., Lintunen, T., & Chatzisarantis, N. L. D. (2012b). An intervention to reduce alcohol consumption in undergraduate students using implementation intentions and mental simulations: A cross-national study. International Journal of Behavioral Medicine, 19 , 82–96. https://doi.org/10.1007/s12529-011-9163-8

Kirk, D., Oettingen, G., & Gollwitzer, P. M. (2013). Promoting integrative bargaining: Mental contrasting with implementation intentions. International Journal of Conflict Management, 24(2), 148–165. https://doi.org/10.1108/10444061311316771

Komesu, Y., Urwitz-Lane, R., Ozel, B., Lukban, J., Kahn, M., Muir, T., Fenner, Dee., & Rogers, R. (2009). Does mental imagery prior to cystoscopy make a difference? A randomized controlled trial. American Journal of Obstetrics and Gynecology, 201 (2), 218e1–218e9. https://doi.org/10.1016/j.ajog.2009.04.008

McNeil, D. G., Spittle, M., & Mesagno, C. (2019). Imagery training for reactive agility: Performance improvements for decision time but not overall reactive agility. International Journal of Sport and Exercise Psychology. Advance online publication. https://doi.org/10.1080/1612197X.2019.1696866

Mendoza, D., & Wichman, H. (1978). “Inner” darts: Effects of mental practice on performance of dart throwing. Perceptual and Motor Skills, 47 (Suppl. 3), 1195–1199. https://doi.org/10.2466/pms.1978.47.3f.1195

Overdorf, V., Page, S. J., Schweighardt, R., & McGrath, R. E. (2004). Mental and physical practice schedules in acquisition and retention of novel timing skills. Perceptual and Motor Skills, 99 , 51–62. https://doi.org/10.2466/pms.99.1.51-62

Peluso, E. A., Ross, M. J., Gfeller, J. D., & LaVoie, D. J. (2005). A comparison of mental strategies during athletic skills performance. Journal of Sports Science and Medicine, 4(4), 543

Raison, N., Ahmed, K., Abe, T., Brunckhorst, O., Novara, G., Buffi, N., McIlhenny, C., van der Poel, H., van Hemelrijck, M., Gavazzi, A., & Dasgupta, P. (2018). Cognitive training for technical and nontechnical skills in robotic surgery: a randomised controlled trial. BJU International, 122(6), 1075–1081. https://doi.org/10.1111/bju.14376

Rawlings, E. I., Rawlings, I. L., Chen, S. S., & Yilk, M. D. (1972). The facilitating effects of mental rehearsal in the acquisition of rotary pursuit tracking. Psychonomic Science, 26(2), 71–73. https://doi.org/10.3758/BF03335435

Renner, F., Murphy, F. C., Ji, J. L., Manly, T., & Holmes, E. A. (2019). Mental imagery as a “motivational amplifier” to promote activities. Behaviour Research and Therapy, 114, 51–59. https://doi.org/10.1016/j.brat.2019.02.002

Ross, S. L. (1985). The effectiveness of mental practice in improving the performance of college trombonists . Journal of Research in Music Education, 33 (4), 221– 230. https://doi.org/10.2307/3345249

Saimpont, A., Lafleur, M. F., Malouin, F., Richards, C. L., Doyon, J., & Jackson, P. L. (2013). The comparison between motor imagery and verbal rehearsal on the learning of sequential movements. Frontiers in Human Neuroscience, 7 , 773. https://doi.org/10.3389/fnhum.2013.00773

Sanders, C. W., Sadoski, M., van Walsum, K., Bramson, R., Wiprud, R., & Fossum, T. W. (2008). Learning basic surgical skills with mental imagery: using the simulation centre in the mind. Medical Education, 42, 607–612. https://doi.org/10.1111/j.1365-2923.2007.02964.x

Seif-Barghi, T., Kordi, R., Memari, A. H., Mansournia, M. A., & Jalali-Ghomi, M. (2012). The effect of an ecological imagery program on soccer performance of elite players. Asian Journal of Sports Medicine, 3 (2), 81. https://doi.org/10.5812/asjsm.34703

Shackell, E. M., & Standing, L. G. (2007). Mind over matter: Mental training increases physical strength. North American Journal of Psychology, 9 (1).

Shanks, D. R., & Cameron, A. (2000). The effect of mental practice on performance in a sequential reaction time task. Journal of Motor Behavior, 32(3), 305–313. https://doi.org/10.1080/00222890009601381

Sheeran, P., Harris, P., Vaughan, J., Oettingen, G., & Gollwitzer, P. M. (2013). Gone exercising: Mental contrasting promotes physical activity among overweight, middle-aged, low-SES fishermen. Health Psychology, 32(7), 802. https://doi.org/10.1037/a0029293

Smith, D., Collins, D., & Holmes, P. (2003). Impact and mechanism of mental practice effects on strength. International Journal of Sport and Exercise Psychology, 1(3), 293–306. https://doi.org/10.1080/1612197X.2003.9671720

Smyth, M. M. (1975). The role of mental practice in skill acquisition. Journal of Motor Behavior, 7 (3), 199–205. https://doi.org/10.1080/00222895.1975.10735034

Strachan, S. M., Marcotte, M. M. E. Giller, T. M. T., Brunet, J., & Schellenberg, B. J. I. (2017). An online intervention to increase physical activity: Self-regulatory possible selves and the moderating role of task self-efficacy. Psychology of Sport and Exercise, 31 , 158–165. https://doi.org/10.1016/j.psychsport.2016.05.001

Theeuwes, M., Liefooghe, B., De Schryver, M., & De Houwer, J. (2018). The role of motor imagery in learning via instructions. Acta Psychologica, 184, 110–123. https://doi.org/10.1016/j.actpsy.2017.05.002

Wohldmann, E. L., Healy, A. F., & Bourne Jr, L. E. (2007). Pushing the limits of imagination: Mental practice for learning sequences. Journal of Experimental Psychology: Learning, Memory, and Cognition, 33(1), 254. https://doi.org/10.1037/0278-7393.33.1.254

Wollman, N., Hill, J., & Lipsitz, T. (1985). Effects of imagery on track and bowling performance in naturalistic settings. Perceptual and Motor Skills, 60 (3), 986–986. https://doi.org/10.2466/pms.1985.60.3.986

Yue, G., & Cole, K. J. (1992). Strength increases from the motor program: Comparison of training with maximal voluntary and imagined muscle contractions. Journal of Neurophysiology, 67(5), 1114–1123. https://doi.org/10.1152/jn.1992.67.5.1114

Zecker, S. G. (1982). Mental practice and knowledge of results in the learning of a perceptual motor skill. Journal of Sport Psychology, 4 (1), 52–63. https://doi.org/10.1123/jsp.4.1.52

Zinatelli, M., & Vogel-Sprott, M. (1990). Learned tolerance to alcohol: Mental rehearsal with imagined consequences. Alcoholism: Clinical and Experimental, 14, 518–521. https://doi.org/10.1111/j.1530-0277.1990.tb01191.x
